# A broad range quorum sensing inhibitor working through sRNA inhibition

**DOI:** 10.1038/s41598-017-09886-8

**Published:** 2017-08-29

**Authors:** Tim H. Jakobsen, Anders N. Warming, Rebecca M. Vejborg, Joana A. Moscoso, Marc Stegger, Frederik Lorenzen, Morten Rybtke, Jens B. Andersen, Rico Petersen, Paal Skytt Andersen, Thomas E. Nielsen, Tim Tolker-Nielsen, Alain Filloux, Hanne Ingmer, Michael Givskov

**Affiliations:** 1 Costerton Biofilm Center, Department of Immunology and Microbiology, University of Copenhagen, Copenhagen, Denmark; 2 Department of Veterinary Pathobiology, University of Copenhagen, Copenhagen, Denmark; 3 Centre for Molecular Bacteriology and Infection, Division of Cell and Molecular Biology, Imperial College London, London, UK; 4 Department of Microbiology and Infection Control, Statens Serum Institut, Copenhagen, Denmark; 5 Department of Chemistry, Technical University of Denmark, Kgs, Lyngby, Denmark; 6 Singapore Centre on Environmental Life Sciences Engineering, Nanyang Technological University, Singapore, Singapore

## Abstract

For the last decade, chemical control of bacterial virulence has received considerable attention. Ajoene, a sulfur-rich molecule from garlic has been shown to reduce expression of key quorum sensing regulated virulence factors in the opportunistic pathogen *Pseudomonas aeruginosa*. Here we show that the repressing effect of ajoene on quorum sensing occurs by inhibition of small regulatory RNAs (sRNA) in *P*. *aeruginosa* as well as in *Staphylococcus aureus*, another important human pathogen that employs quorum sensing to control virulence gene expression. Using various reporter constructs, we found that ajoene lowered expression of the sRNAs RsmY and RsmZ in *P*. *aeruginosa* and the small dual-function regulatory RNA, RNAIII in *S*. *aureus*, that controls expression of key virulence factors. We confirmed the modulation of RNAIII by RNA sequencing and found that the expression of many QS regulated genes encoding virulence factors such as hemolysins and proteases were lowered in the presence of ajoene in *S*. *aureus*. Importantly, our findings show that sRNAs across bacterial species potentially may qualify as targets of anti-virulence therapy and that ajoene could be a lead structure in search of broad-spectrum compounds transcending the Gram negative-positive borderline.

## Introduction

Both the Gram-negative *Pseudomonas aeruginosa* and the Gram-positive *Staphylococcus aureus* can cause acute as well as chronic infections. They are the cause of a number of nosocomial infections in primarily immune-compromised individuals^1–3^ as well as people with leg and foot ulcers, and are among the most common airway pathogens in patients with the genetic hereditary disease cystic fibrosis (CF)^4^. Both are versatile pathogens relying on a diverse array of virulence factors to colonize and infect hosts and their capability, in the form of biofilms, to evade host immune systems^5, 6^. New intervention strategies are in a great demand due to an increasing resistance development in *P*. *aeruginosa* and *S*. *aureus* strains to conventional antibiotics and inherent difficulties in treating biofilm-associated infections^7^. One antimicrobial strategy, which has gained attention is small molecules perturbations of the cell-cell communication system termed quorum sensing (QS) by *i*.*e*. QS inhibitors (QSIs). *P*. *aeruginosa* and *S*. *aureus* can often be found in polymicrobial infections with chronic wounds being the most prevalent example^8^. A number of studies investigate potential interaction between these two organisms and the relevance to infection development and treatment options^9^.

The QS systems have been intensively studied in both Gram-positive and Gram-negative pathogens over the last decades. Many inhibitors of each of the systems have been identified over the years, whereas identification of common inhibitors of both the Acyl-Homoserine Lactone (AHL) system in Gram-negative and the Autoinducing Peptide (AIP) system in Gram-positive are uncommon. Although the pathways and signalling mechanisms of the two systems are very distinct important similarities do exist. In both *P*. *aeruginosa* and *S*. *aureus*, sRNAs play a key role in regulating the cellular responses to the signal molecules. In *P*. *aeruginosa*, two sRNAs, RsmY and RsmZ, have been shown to have high affinity for the global regulator protein RsmA that in its free form represses translation of a number of genes including QS target mRNAs by preventing ribosome binding to the Shine-Dalgarno (SD) sequences^10^. RsmA modulates the expression of several important functionalities including virulence factors, polysaccharides and motility^11, 12^. In *S*. *aureus*, the sRNA, RNAIII is the effector of QS-induced gene expression encoded by the *agr* locus. It regulates gene expression either directly by RNA:RNA interaction as in the case of Protein A (*spa)* and α-hemolysin (*hla*)^13, 14^ or indirectly by controlling the transcript levels of other regulators such as the Repressor of Toxins (Rot)^15^. Abolishing RNAIII expression in stationary phase (where expression usually peaks as a consequence of increased cell density^16^) has profound effects on the transcriptome including prevention of expression of major virulence factors such as α-hemolysin, lipase and proteases which are normally produced abundantly when RNAIII is expressed^17^.

A two-component transduction system governs control of sRNA synthesis in both *P*. *aeruginosa* and *S*. *aureus*. Transcription of *rsmY* and *rsmZ* in *P*. *aeruginosa* is positively regulated by the two-component transduction system denoted Gac (Global activator of Antibiotic and Cyanide synthesis)^10, 18, 19^. RsmY and RsmZ sequester RsmA, but in their absence, RsmA represses translation of Pel and Psl exopolysaccharides and the type VI secretion system (T6SS)^11, 20^. In contrast, RsmA positively regulates type IV pili and type III secretion system (T3SS)^20^. For this reason, the Gac/Rsm system is believed to govern the switch between the planktonic and the biofilm mode of growth of *P*. *aeruginosa*, and to coordinate a switch between an acute and a chronic mode of infection^21^. Important to the Gac/Rsm regulatory network are also the two sensors RetS and LadS, which impact the phosphorylation status of GacS^22–24^. When RsmY and RsmZ are present in high concentrations, RsmA is sequestered and this allows the production of the two AHL signal molecules, C4-HSL and 3-oxo-C12-HSL^25, 26^. In *S*. *aureus*, the *agr* encoded complex consists of four peptides, AgrA, AgrB, AgrC and AgrD. AgrC is a two-component histidine kinase sensor, which responds to the AIPs binding by autophosphorylating^27^ and subsequently transferring the phosphate group to AgrA, causing a conformational change. Phosphorylated AgrA (and, to a lesser degree, unphosphorylated AgrA) binds to two sets of direct repeats in the region between the *agr* P2 and P3 (RNAIII) promoters and activates transcription from both promoters^28, 29^.

Many herbs produce QSI molecules and recently, we identified ajoene to be the major QSI present in garlic^30^. Ajoene is a small sulfur-rich molecule produced from two allicin molecules, which originate from aliin by an enzymatic process that occurs when garlic is crushed or chopped. Our previous studies with synthetic ajoene revealed a molecule capable of blocking the QS-regulated production of rhamnolipid in *P*. *aeruginosa*, resulting in polymorphonuclear leucocytes (PMNs) being able to phagocytose biofilms and lower the resistance of *in vitro* and *in vivo* biofilms to aminoglycosides including tobramycin^30, 31^. In addition, *in vivo* studies showed a significant reduction of pulmonary infection in ajoene-treated mice compared to untreated^30^. In the present study, we deliver experimental evidence showing that ajoene mediates its QS repressing effect through modulation of sRNA expression of *rsmY* and *rsmZ* in *P*. *aeruginosa* as well as presenting QS inhibitory activity of ajoene against *S*. *aureus* by showing modulation of *rnaIII* expression and other important QS regulated genes.

## Results

### Decrease of *rsmY* and *rsmZ* expression by ajoene in *P*. *aeruginosa*

**Figure 1:**
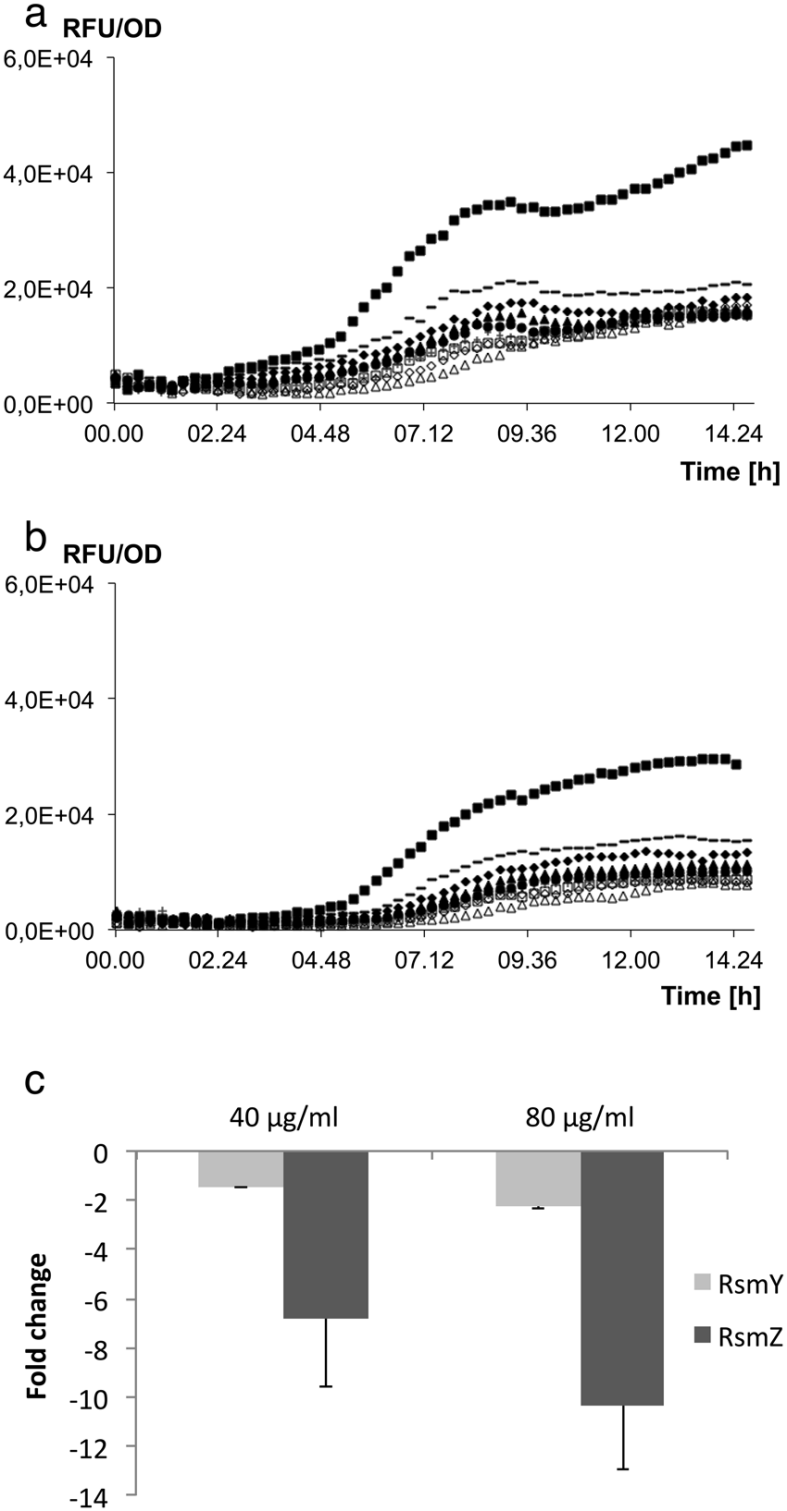
Regulation of *rsmY* and *rsmZ* production by ajoene. The GFP expression/cell density (RFU/OD) of a *rsmY-gfp* (**a**) and *rsmZ-gfp* (**b**) transcriptional fusion with following concentrations of ajoene: △125 μg/ml ◊62,5 μg/ml □31,25 μg/ml +15,6 μg/ml ●7,8 μg/ml ▲3,9 μg/ml ♦2 μg/ml −1 μg/ml ■ No addition of ajoene. The experiments were done in triplicate. (**c**) qRT-PCR measurements of fold changes in *rsmY* (light grey bars) and *rsmZ* (dark grey bars) contents at early stationary growth phase (OD_600_ of 2.0). The data represent average of three individual experiments. Error bars are means ± SDs.

In a previous study, we speculated, based on a gene-chip based transcriptomic analysis, that the QS inhibitory effect of ajoene on *P*. *aeruginosa* could be exerted upstream of the central QS machinery, notably by targeting the Gac/Rsm cascade. In the same study we measured a decrease in expression with our QSI reporter systems, *lasB-gfp* and *rhlA-gfp* which are translational constructions supporting the possibility that ajoene targets the Gac/Rsm cascade^30^. In the present study we used *rsmY-gfp* (PAO099) and *rsmZ-gfp* (PAO098) transcriptional fusions to demonstrate that exposure to ajoene decreased expression of the sRNAs in a dose-dependent manner (Fig. 1a,b), with half-maximal inhibitory concentrations (IC_50_) for *rsmY* on 2.5 µg/ml (10.7 μM) and *rsmZ* on 2.3 µg/ml (9.8 μM), respectively (Fig. S1). The Minimal Inhibitory Concentration (MIC) of the synthetic ajoene used in this study was >160 µg/ml against wild type (*wt*) PAO1 and 20 µg/ml against *wt S*. *aureus* 8325-4, which corresponds to the results shown in a earlier study of the antimicrobial activity of ajoene^32^. The decrease in expression of *rsmY* and *rsmZ* by ajoene was further validated by qRT-PCR. Cultures with *wt* PAO1 were treated with 40 μg/ml (170.8 μM) and 80 μg/ml (341.6 μM) ajoene and retrieved at late exponential and early stationary growth phase (Fig. S2). There was a limited inhibition of *rsmY* and *rsmZ* expression at late exponential phase as expected due to the low expression of *rsmY* and *rsmZ* early in the growth phase, whereas at early stationary phase, expression of *rsmY* and *rsmZ* was reduced 4- and 10-fold, respectively, in the sample with 80 μg/ml ajoene administered (Fig. 1c).

### Ajoene’s QS inhibition activity depends on *rsmY* and *rsmZ*

Ajoene did neither affect *lasB* expression in the *rsmY* (PAO102) and *rsmZ* (PAO103) single mutants nor in an *rsmYZ* double mutant (PAO101) (Fig. S3a–c). This loss of QS inhibitory activity taken together with the inhibitory effect of *rsmY* and *rsmZ* expression in the *wt* background indicated that ajoene exerts its action on QS through the Gac/Rsm cascade by decreasing the synthesis of RsmY and RsmZ. The lack of impact of ajoene in the single mutant backgrounds illustrates that both sRNAs need to be present to effectively sequester RsmA. The impact of ajoene on *lasB* expression in an *rsmA* mutant (PAO104) was also tested. However, the growth inhibitory activity of ajoene was too high in this particular mutant strain to generate any useable results (Fig. S3e). In a *gacA* mutant (PAO105), *lasB* expression was to low to measure any QS inhibitory activity of ajoene (Fig. S3d).

The effect of ajoene on *rsmY* and *rsmZ* expression could be redily explained if ajoene worked as a kinase inhibitor that would subsequently affect the phosphorylation state of GacA. However, we were unable to record any change in GacA phosphorylation by using either a GacA antibody combined with SuperSep Phos-tag gels or Phos-tag phosphorprotein gel stain (Fig. S4). Moreover, so far we have not been able to detect any direct molecular interaction between the sRNAs and ajoene in a gel mobility shift assay suggesting that ajoene is not likely forming a complex with the sRNAs (data not shown). To investigate whether ajoene has a general impact on gene transcription modulation of additional sRNAs (*phrS, prrF* and *prrH*) and the housekeeping genes *rpoD* were studied by qRT-PCR (Fig. S5). A small increase in transcript levels of *phrS, prrF* and *prrH* and a 0.6 fold decrease of *rpoD* transript levels in cultures with the addition of 80 μg/ml ajoene indicate that ajoene does not generate a general impact on transcript levels.

**Figure 2:**
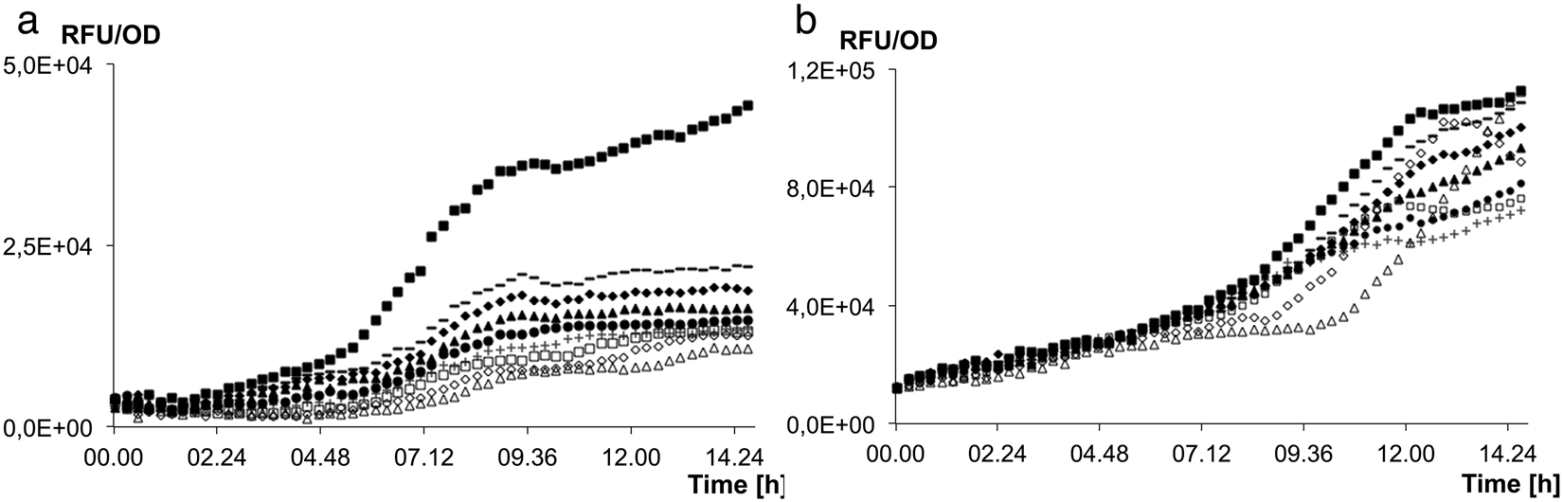
Regulation of *rsmY* and *rsmZ* expression in a *retS* mutant by ajoene. The GFP expression/cell density (RFU/OD) of a *rsmY-gfp* (**a**) and *rsmZ-gfp* (**b**) transcriptional fusion in *retS* background with following concentrations of ajoene: △125 μg/ml ◊62,5 μg/ml □31,25 μg/ml +15,6 μg/ml ●7,8 μg/ml ▲3,9 μg/ml ♦2 μg/ml −1 μg/ml ■ No addition of ajoene. The experiments were done in triplicate.

In a *retS* mutant, ajoene inhibited *rsmY* expression (PAO107) to the same extent as in the *wt*, whereas, ajoene showed minor inhibitory activity on *rsmZ* expression (PAO106) (Fig. 2). This indicates that ajoene does not mediate its effect through RetS. The difference in inhibition by ajoene of *rsmY* and *rsmZ* expression in a *retS* mutant suggests that the inhibitory activity of ajoene potentially involves interaction with several targets.

### Ajoene changes Gac controlled phenotypes

To assess the efficacy of ajoene to modulate phenotypes under the control of the Gac/Rsm cascade we investigated changes in the expression level of T3SS and T6SS, PSL production, biofilm formation as well as eDNA production. Our results suggest that I) ajoene increases T3SS protein production and decreases T6SS protein production in a growth medium-dependent manner, II) modulates the transition between motility and biofilm shown by a decrease in biofilm mass in a static biofilm system, III) decreases the production or release of eDNA in a static biofilm with increasing non-growth inhibitory concentrations, and finaly IV) decreases polysaccharide production shown by Congo red staining (Fig. S6).

### Impact of ajoene on virulence gene expression and aggregation of *S*. *aureus* 8325-4

**Figure 3:**
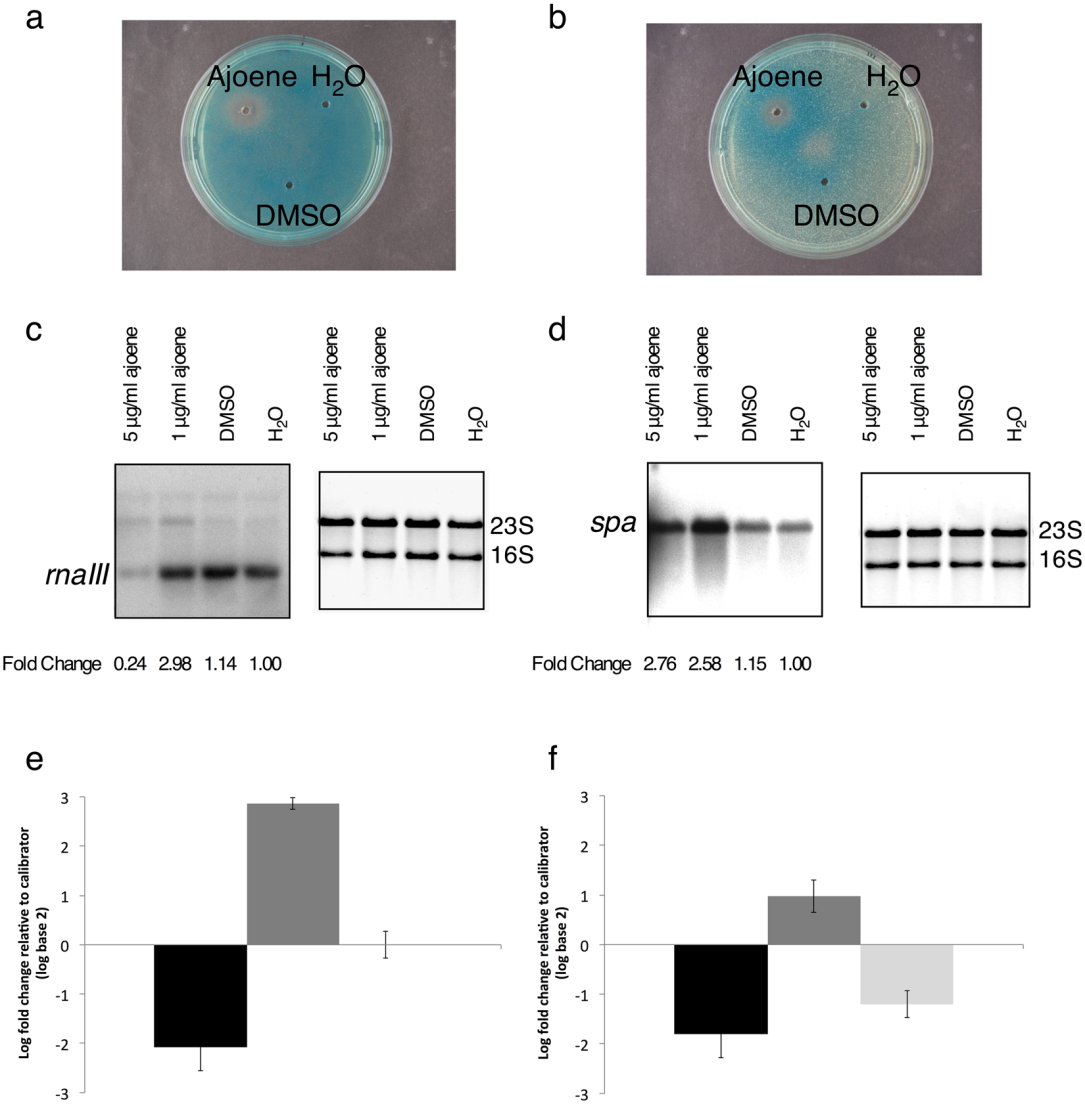
Changes in expression of *rnaIII* and *spa* by ajoene. Expression measured by reporter fusion based plate assay (**a**,**b**), Northern blot (**c**,**d**) and qRT-PCR (**e**,**f**). (**a**) Expression of *rnaIII* measured with *rnaIII::lacZ* reporter fusion. (**b**) Expression of *spa* measured with *spa::lacZ* reporter fusion. (**c**) Expression of *rnaIII* and (**d**) expression of *spa* with the addition of 1 μg/ml and 5 μg/ml of ajoene compared to controls with addition of DMSO or H_2_O. The right panels in (**c**,**d)** show the loading controls of ethidium bromide stained total RNA. (**e**) qRT-PCR data of samples retrieved at exponential growth phase and, (**f**) at stationary growth phase for *rnaIII* (dark grey bars), *spa* (medium grey bars) and *hla* (light grey bars).

In addition to our studies of the effect of ajoene on sRNAs in *P*. *aeruginosa*, we investigated whether ajoene could affect QS in other microorganisms known to utilize sRNAs in the regulation of virulence. To that end, we examined the effect of ajoene on *S*. *aureus*. As an initial screen, we used transcriptional reporter gene fusions to virulence factors in a diffusion-based well plate assay^33^. We found that in the presence of ajoene, expression of *rnaIII* was decreased and expression of *spa* was increased (Fig. 3). In order to determine the effect of ajoene on growth of *S*. *aureus* 8325-4, cultures were grown in the presence of increasing concentrations of ajoene. At concentrations of 1–5 μg/ml, no effect on growth was observed, but increasing the concentration further caused a temporary bacteriostatic effect, with lag phase increasing in a dose dependent manner (Fig. S7). Cell aggregates began to appear in untreated cultures around an optical density (OD_600_) of 2 and could be observed macroscopically as biofilm growth on the surface of the test tube and clumbs of cells in the flasks at an OD_600_ of 3 as has been observed for *S*. *aureus* 8325-4 previously^34^. However, no aggregation or biofilm attached to the test tube was observed in cultures treated with ajoene in concentrations of 1 μg/ml or higher. This is reflected in the shape of the growth curves in Fig. S7; the curves of the untreated cultures became erratic and flatten out around an OD_600_ of 2 due to the fact that aggregation interferes with measuring OD_600_, whereas the cultures treated with 1, 3 and 5 μg/ml of ajoene continued to increase in OD values. This prompted us to investigate a potential effect of ajoene on *S*. *aureus* biofilm development. From a static biofilm experiment no difference in total biofilm mass after 24 hours of growth with non-growth inhibitory concentrations of ajoene was measured when compared to a non-treated control (Fig. S8).

The effect of ajoene on expression of *rnaIII* and *spa* was confirmed with Northern blots (Fig. 3c,d) and qRT-PCR (Fig. 3e,f). With the addition of 5 μg/ml ajoene, a clear decrease in *rnaIII* expression was monitored by Northern blot and with 1 μg/ml a clear increase in *spa* expression were monitored compared to the negative controls. The qRT-PCR data showed the highest decrease in *rnaIII* expression and highest increase in *spa* expression in exponential growth phase (Fig. 3e), whereas the highest decrease in *hla* (encoding α hemolysin) was detected in stationary growth phase (Fig. 3f). When comparing the three experimental methods the same trend was monitored with repression of *rnaIII* and increase of *spa*.

### Effects of ajoene on the transcriptome of *S*. *aureus* 8325-4

To obtain further insight into the QS inhibitory activity of ajoene on *S*. *aureus* we investigated how ajoene influences the transcriptome of *S*. *aureus* 8325-4 by RNA sequencing. *S*. *aureus* cultures were treated with 5 μg/ml ajoene; a concentration that did not interfere with growth rate (Fig. S7a,b), and samples were retrieved at mid exponential, late exponential and early stationary growth phase, which corresponds to 5′, 60′ and 240′ minutes after addition of ajoene, respectively.

Different microarray analyses of the *agr*-regulated transcriptome in *S*. *aureus* have identified core virulence factors that depend on either AgrA or RNAIII regulation. However, these studies investigate the transcriptome of other *S*. *aureus* strains than 8325-4 and do not show complete consistency between QS regulated genes^17, 35^. Nevertheless, Table 1 has been generated from these studies displaying genes that are supposedly up- or down-regulated by QS and more specifically by RNAIII. Here we show a very good accordance between QS regulated genes in *S*. *aureus* and the genes affected by ajoene treatment (Table 1).

**Table 1:**
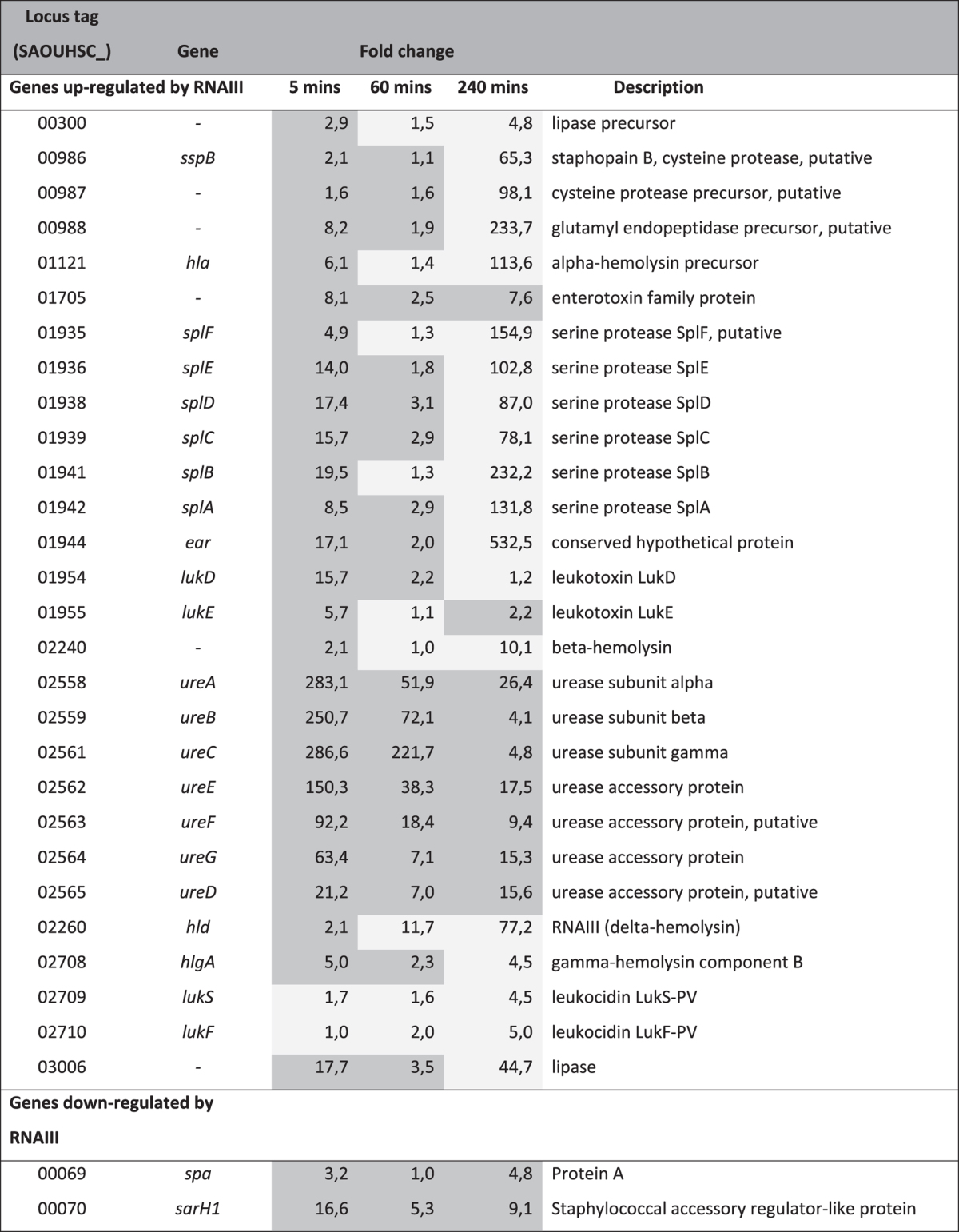
Alteration of *S*. *aureus* 8325-4 gene expression by ajoene. Genes displayed in the table are regulated by RNAIII^a^. ^a^Light grey: genes down-regulated by ajoene. Dark grey: genes up-regulated by ajoene.

At 240’ minutes after addition of ajoene, expression of a range of QS regulated genes encoding virulence factors were down regulated. In particular, *hld* encoding RNAIII was down regulated 77-fold as well as the genes *hla*, SAOUHSC_02240, SAOUHSC_02708 (encoding α, β and γ-hemolysin), *splA-F* and *sspABC* (encoding the secreted serine- and cysteine proteases) and SAOUHSC_03006 and SAOUHSC_00300 (encoding lipases) were all down regulated more than 5-fold compared to the untreated cultures. The genes *spa* and *sarH1*, encoding protein A and Staphylococcal accessory regulator-like protein are both up-regulated in the culture treated with ajoene, which corresponds to the indications from the literature that the genes are down-regulated by RNAIII.

To verify our RNA sequencing data set, we compared the observed changes in 8325-4 gene expression during growth in presence of ajoene to previously described gene expression patterns. The P2 and P3 transcripts from the *agr* operon follow the well-established QS-induced transcription pattern, a low transcript level in exponential phase and increasing as growth progressed (Table S1)^17^. The increase in P2 transcript (encoding *agrABCD*) during the experiment was much smaller (6-fold increase) than the increase in P3 (RNAIII) transcript levels (300-fold increase), similar to what has previously been observed^36^. As would be expected given the rise in *agr* P2 and P3 transcript levels^16^, we saw an increase in *splA-F* and *sspABC* transcript levels, (encoding the secreted serine and cysteine proteases, respectively) at time 240. *sarA* transcription levels decreased between 5′ and 60′ minutes and very little transcript remained at 240′ minutes, similar to what has previously been found in 8325-4^37^. On the basis of these observations, we conclude that the RNA sequencing data set is useful in assessing changes in transcript levels in our samples.

## Discussion

A strategy that target expression of virulence factors and not bacterial growth could be of benefit in relation to the chemotherapeutic resistance we have seen for decades with the conventional antibiotic therapy. The key to success for *P*. *aeruginosa* and *S*. *aureus* as pathogens are their well-developed mechanisms of immune evasion including their capacity of biofilm formation that mitigates the impact of antimicrobial strategies based on conventional antibiotics. Antivirulence strategies that reinstate proper action of the cellular immune system and conventional antibiotics could therefore potentially become a stepping-stone in the design of future, chemotherapeutic treatment regimens.

Our present results indicate that the target of ajoene in *P*. *aeruginosa* is the Gac/Rsm part of the QS machinery which results in low expression of the two small regulatory RNAs, *rsmY* and *rsmZ* leading to a increase in the post-transcriptional negative effect of RsmA on *lasI* and *rhlI* expression. Several studies have investigated and elucidated the role of RsmA on several important factors contributing to the pathogenicity of *P*. *aeruginosa*. We found that ajoene has an effect on several of these factors by phenotypic investigations indicating that it is possible to modulate the influence of RsmA by regulating the expression of *rsmY* and *rsmZ*.

In our previous study we found that ajoene down-regulates a subset of important virulence factors in *P*. *aeruginosa* including rhamnolipids^30^ known for the effect of killing PMNs among others^38^. In addition we found that a *P*. *aeruginosa* biofilm treated with ajoene is more sensitive to aminoglycosides than the untreated biofilm. It is known that a decrease in Psl and Pel as well as a lowered content of eDNA tend to destabilize biofilms^39^. Our present results showed a decrease in both Psl and eDNA in a *P*. *aeruginosa* culture treated with increasing concentrations of ajoene, which corresponds to our data showing a decrease in biofilm mass in a static biofilm model, likely by sloughing-off due to mechanical shearing produced during the assay. Whilst ajoene does not inhibit biofilm formation *per se*, ajoene exposure destabilizes the biofilm structure. Besides its structural contribution eDNA increases the tolerance of a biofilm to aminoglycosides^40^. The decrease in eDNA content in a *P*. *aeruginosa* biofilm caused by ajoene treatment reinstates the sensitivity of the biofilm to aminoglycosides. All together our results explain the observations that *P*. *aeruginosa* biofilms will become more prone to eradication by the immune defence and/or applied antibiotics when treated with ajoene^30, 31^.

The ajoene concentrations used to reach the highest inhibition of *P*. *aeruginosa* QS regulated genes were 16 times higher than for *S*. *aureus*. A similar large difference in ajoene concentration between the two organisms was also seen in relation to MIC values. The difference in effective concentration of ajoene towards the two organisms may potentially be generated by the general difference in cell membrane permeability between Gram-positive and Gram-negative bacteria.

In this study, we present evidence of a decrease in *rnaIII* expression and increase in *spa* expression in *S*. *aureus* in response to ajoene exposure. The global impact of this was established by means of *S*. *aureus* RNA sequencing data that showed the transcript levels of RNAIII, expression of *hla* and the genes encoding lipase and all of the extracellular proteases did not increase upon entry into stationary phase in the presence of ajoene. Ajoene seems to cause a transient and moderate increase in transcript levels of secreted factors after being added in early exponential phase, but to cause a severe decrease in transcript levels of many secreted factors later in the transition to stationary phase. The expected increase in transcript levels of hemolysins, proteases and lipases in stationary phase was not observed when ajoene was added, and a similar effect on transcript levels of the Ess system was observed at 5′ and 60′ minutes. The Ess system was originally investigated in *Mycobacterium tuberculosis*, where it was found to be important for establishing tuberculosis by allowing *M*. *tuberculosis* to replicate in macrophages^41^. It was recently found to be important for abscess formation and persistence during an *S*. *aureus* infection^42, 43^ and is now the topic of much attention. Consequently, the Ess represents an interesting target for anti-virulence treatment.

The transcript levels of some secreted factors (PSM-α (phenol-soluble modulin α) and Panton-Valentine Leukocidin (PVL) encoded by *lukS-PV* and *lukF-PV*, which are controlled by the QS system were not affected. This is not surprising since very little transcript was detected for these factors in our strain under the growth conditions used and the times sampled, similarly to all enterotoxins.

**Figure 4:**
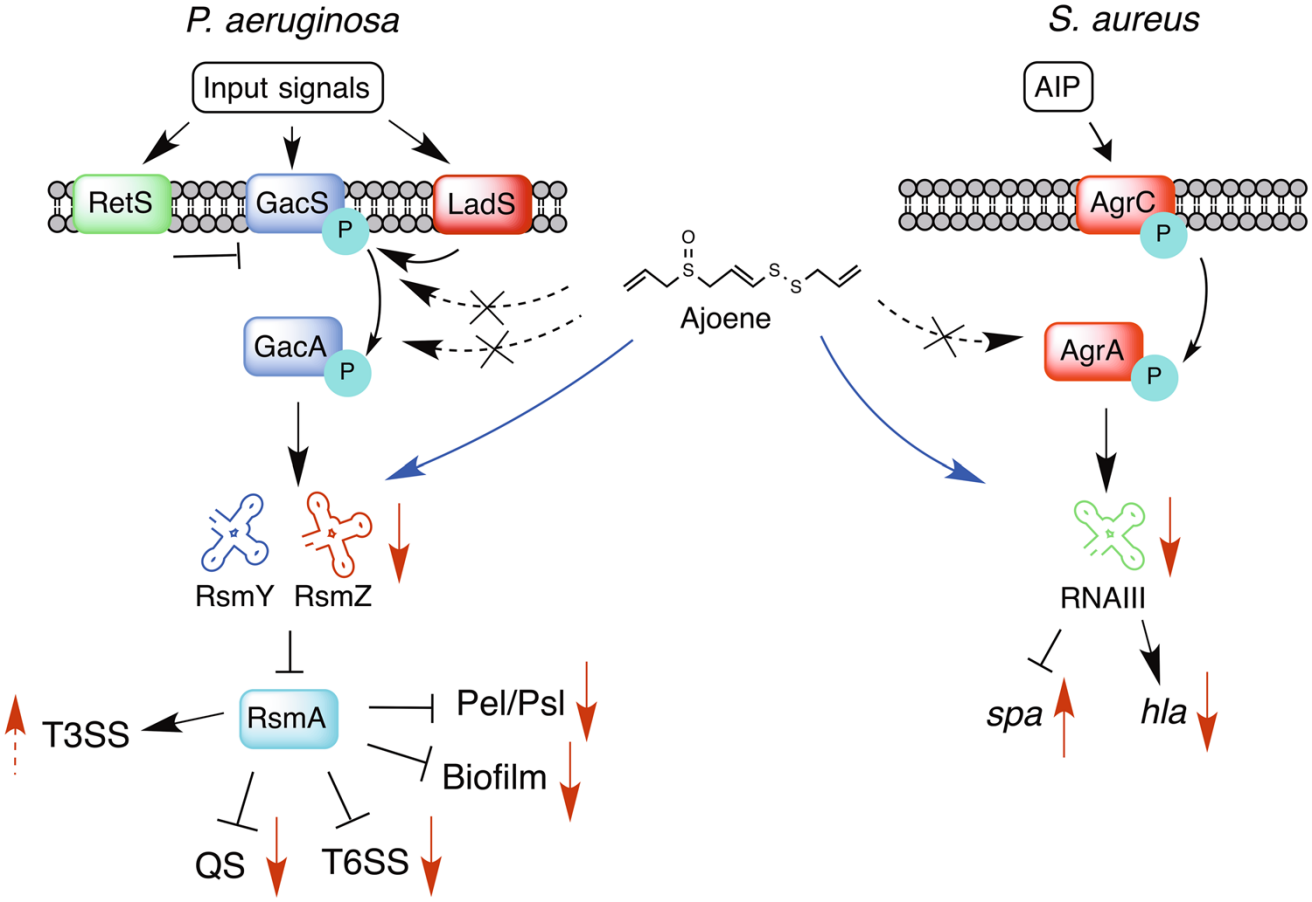
Regulatory effects of ajoene in *P*. *aeruginosa* and *S*. *aureus*. Simplified overview of the two-component regulatory systems in *P*. *aeruginosa* and *S*. *aureus* controlling the expression of the sRNAs. In *P*. *aeruginosa* GacS/GacA induces expression of the two sRNAs, RsmY and RsmZ and in *S*. *aureus* AgrC/AgrA induces expression of the sRNA RNAIII. The decrease in expression of *rsmY* and *rsmZ* is believed to increase free RsmA leading to changes in different phenotypic traits. Red arrows, measured effect of ajoene; dashed red arrows, possible effect of ajoene; black dashed arrows with cross, unlikely target of ajoene; blue arrows, effect of ajoene on regulatory systems presumably by non-directly interaction; black arrows, positive control in the Gac/Rsm cascade and Agr system; T-bars, negative control in the Gac/Rsm cascade and Agr system.

The alterations in transcript levels in *S*. *aureus* caused by ajoene seems not to be due to changes in *agr* P2 expression, since the P2 transcript only showed a 2-fold or smaller decrease compared to the untreated cultures, a magnitude that cannot be assumed to be biologically meaningful due to the technical variation observed, suggesting that the observed effect of ajoene on RNAIII transcript levels was independent of the P2 transcript. In addition, ajoene-mediated inhibition of QS regulated genes in *S*. *aureus* and *P*. *aeruginosa* does not appear to be the result of a general inhibition of transcription. In Fig. 4, a simplistic model is illustrating the effects of ajoene and potential targets on *P*. *aeruginosa* and *S*. *aureus*. Given the complex, transcriptome-wide effect observed in the *S*. *aureus* treated cells, cause and effect could not be fully elucidated from the current data and it remains unknown how the effects of ajoene on RNAIII transcript levels is mediated. Our data does not indicate direct interaction between ajoene and the sRNAs. Our hypothesis is that ajoene targets regulatory pathways affecting expression of the RNAs involved in the QS regulatory systems with high specificity and not by a general impact on transcription. Our sRNA *P*. *aeruginosa* reporter constructs show a similar level of inhibition of both *rsmY* and *rsmZ* expression by ajoene, whereas our qRT-PCR data show a larger inhibition of *rsmZ* compared to *rsmY*, and in a *retS* mutant a lower inhibition of *rsmZ* is measured. This indicates that the different conditions used in each of the experiments generate a difference in inhibition of the two sRNAs.

## Conclusion

We have shown that ajoene is mediating its QSI activity by lowering the expression of sRNAs in both *P*. *aeruginosa* and *S*. *aureus*. To date reported QSIs are generally highly species specific. Ajoene is one of a few examples of a broad-spectrum range QSIs and the first compound to be reported to target QS by sRNA modulation in both Gram-negative and Gram-positive bacteria. The efficacy as antimicrobial compound in animal models of infection puts focus on targets that modulate sRNA in antibacterial drug development, which as an example could be the GacS/GacA and AgrC/AgrA two-component systems in *P*. *aeruginosa* and *S*. *aureus*, respectively. However, from our study the decrease in expression of *rsmY* and *rsmZ* by ajoene does not appear to be generated by kinase inhibition of GacS/GacA. This is in correlation with the transcriptomic analysis of ajoene treated *S*. *aureus* cultures which indicate that the reduction in RNAIII transcript is not generated by kinase inhibition of AgrC/AgrA because no reduction in the *agr* locus was observed.

Strategies to identify new QSIs against especially the AHL QS system have been particular focused on AHL structural analogues targeting the central parts of the QS system. However, this study with ajoene and previous studies with iberin^44^ and azithromycin^45^ have shown that sRNAs are a potential target to alter the expression of QS controlled virulence factors and phenotypic traits under the control of RsmA. This is also valid for *S*. *aureus* in which a number of studies have shown the effect on virulence by for instance reducing RNAIII by blocking AgrA binding^46^. These results need further investigations, not least in terms of identifying the specific mechanisms of action leading to impact on RsmY and RsmZ in *P*. *aeruginosa* as well as on RNAIII in *S*. *aureus*.

## Materials and Methods

### Bacterial strains, plasmids and growth conditions

The bacterial strains and plasmids used in this study are listed in Table S2. The medium used for *P*. *aeruginosa* was either Luria-Bertani (LB) medium or AB minimal medium (B medium^47^ plus 10% A10^47^) supplemented with 0.5% (wt/vol) glucose and 0.5% (wt/vol) Casamino Acids for growing the monitor strains (overnight (O.N.) cultures). All *S*. *aureus* strains were grown in tryptic soy broth (TSB, Oxoid), supplemented with agar when solid medium was needed (yielding tryptic soy agar, TSA). Unless otherwise stated, all strains were incubated at 37 °C with shaking (200 rpm) and supplemented with antibiotics where appropriate.

### Construction of the rsmZ-gfpmut3b* and rsmY-gfpmut3b* transcriptional fusions

The rsmZ-gfpmut3b* and rsmY-gfpmut3b*-based monitor plasmids pRV59_1 and pRV60_1 were constructed by subcloning the *rsmY* and *rsmZ* promoter regions of pMP220*rsmZ-lacZ* and pMP220*rsmY-lacZ* ^48^ into pMH305^49^, a pUCP22NotI-based expression vector carrying a promoterless gfpmut3b* gene^50^ (pMH305 carries the RBSII-*gfp*(Mut3)-T_0_-T_1_ fragment of pJBA25^51^ in the NotI site^49^). The promoter regions were excised by digestion with *KnpI* and *EcoRI* and inserted into *KnpI*/*EcoRI*-digested pMH305, generating rsmZ- gfpmut3b* and rsmY-gfpmut3b* transcriptional fusions. The ligation mixtures were transformed into *E*. *coli* K-12 and selected on selective plates containing 100 μg/ml ampicillin. The plasmid constructs were verified by restriction analysis and subsequently moved into *P*. *aeruginosa* by electroporation and plating on 30 μg/ml gentamicin^52^.

### Construction of *P*. *aeruginosa* ΔretS mutant

The *retS* deletion vector (pΔretS) was constructed following the Gateway-based gene replacement method of Choi and Schweizer^53^ using primers listed in Table S2. Briefly, upstream and downstream regions flanking the part of *retS* to be deleted were amplified using the primer pairs retS_UpF-GWL/retS_UpR-Gm and retS_DnF-Gm/retS_DnR-GWR, respectively. The FRT-flanked gentamicin resistance cassette to replace the *retS* gene was amplified from pPS856 using the primer pair Gm-F/Gm-R. The gene deletion cassette was then assembled from the three fragments by SOE-PCR using the primer pair GW-attB1/GW-attB2 and inserted into the Gateway donor vector pDONR221 using BP clonase (Invitrogen). The resulting intermediate entry vector pENTRretS was finally transferred to the gene replacement vector pEX18ApGW using LR clonase (Invitrogen) thereby creating the *retS* deletion vector pΔretS. The *∆retS* mutant, defective for RetS was constructed by allelic displacement as previously described^53^. Primers and plasmids used for construction of the *retS* deletion mutant are listed in Table S2.

### Chemically synthesized ajoene

Ajoene was synthesized from commercially available distilled allyl disulfide as a 1:4 mixture of E and Z isomers as described by Givskov^54^. Synthetic ajoene was purified by silica gel chromatography and characterized by ^1^H NMR, ^13^C NMR, and HRMS. The purity was greater than 98%. Synthetic ajoene was used in all experiments described in this article.

### MIC evaluation of ajoene

Broth micro-dilution was used to determine MIC values of ajoene against *P*. *aeruginosa* PAO1 and *S*. *aureus* 8325-4. Ajoene were tested in two fold concentrations from 160 μg/ml to 0.31 μg/ml in Mueller-Hinton broth. Start inoculation were 1:100 dilution off O.N. cultures following incubation for 24 hours in 37 °C. MIC values were determined by visual inspection.

### Inhibition assays

#### *P*. *aeruginosa*

The bioassays were performed in 96-well microtiter dish (Black Isoplate; Perkin Elmer) as previously described^30^. The growth of the bacterial cells (OD_450_) and green fluorescent protein (GFP) expression (excitation wavelength, 485 nm; emission wavelength, 535 nm) were measured on a multilabel plate reader (Wallac 1420 VICTOR2; Perkin Elmer) every 15 minutes over 14 hours. The temperature was held constant at 34 °C. IC_50_ values were calculated by means of the software PRISM (GraphPad) from the values of specific fluorescence (GFP/OD) obtained throughout the growth cycle.

#### *S*. *aureus*

The plate assay was performed as previously described (28). Cultures of reporter strains with either the RNAIII or spa promoter fused to a *lacZ* gene were cast into semi-solid TSA agar containing 150 μg/ml 5-bromo-4-chloro-3-indolyl-β-D-galactopyranoside (X-Gal) and 5 μg/ml erythromycin (Sigma-Aldrich). Holes were punched sterilely in the agar, and 20 μl of 500 μg/ml ajoene dissolved in DMSO was added to the designated holes alongside holes containing DMSO and water as controls. Based on the color changes of the areas around the holes, we assessed promoter activity of the two genes when exposed to ajoene.

### RNA preparation

#### *P*. *aeruginosa*

Exponential growing (OD_600_ of 0.5) *P*. *aeruginosa* PAO1 at 37 °C, 180 rpm in AB-media supplemented with 0.5% Casamino acid were diluted to an OD_600_ of 0.1. When reaching OD_600_ of 0.5 the culture were divided and the following concentrations of ajoene were added; 40 µg/ml and 80 µg/ml and one culture with no addition of ajoene. At late exponential growth (OD_600_ of 1.0) and early stationary growth phase (OD_600_ of 2.0) samples were retrieved and two volumes of RNAlater (Ambion) were added. Isolation of RNA was performed using the RNeasy Mini Purification Kit (Qiagen) according to the manufacturer’s instructions and repeated three times with RNA from three individual growth experiments.

#### *S*. *aureus*

Fresh cultures were inoculated from the O.N. cultures and grown in unbaffled flasks with a starting volume:medium ratio of 10:1 at 185 rpm, 37 °C in a water bath. The cultures were inoculated to a starting OD_600_ of 0.01. Upon reaching OD_600_ = 0.2, ajoene dissolved in DMSO was added to a final concentration of 5 μg/ml. Untreated cultures were supplemented with an equal amount of DMSO. After 5′, 60′ and 240′ minutes of growth, culture samples were removed, cooled briefly in ice water and the cells were spun down at 1 °C, 2 minutes, 6797 rcf. Immediately thereafter, the medium was removed and the cells were frozen at −80 °C until the RNA was extracted. The cells were opened using a bead-beating method (FastPrep 24, MP Bio) and total RNA was purified using the RNeasy Mini Purification kit (Qiagen) according to the manufacturer’s instructions. The integrity of the RNA was tested on a Bioanalyzer 2100 using the Prokaryote Total RNA Nano kit (both from Agilent Technologies). All RNA Integrity Numbers (RIN) were over 9.7, indicating that the RNA was intact. The RNA was shipped to Beijing Genomic Institute (BGI) (Hong Kong) on dry ice for rRNA depletion, library construction and sequencing using HiSeq. 2000 technology. Following sequencing, the dataset was filtered for bad reads. The resulting data set consisted of 6 million 49-bp reads per sample.

### qRT-PCR

#### *P*. *aeruginosa*

cDNA was made from 1 μg of RNA using high-capacity RNA-to-DNA master mix (Applied Biosystems). Amplification was performed with SYBR green master mix in a Step One Plus thermal cycler (Applied Biosystems). The primers were designed using Primer Express software. Forty cycles were run with denaturation at 95 °C for 15 s, annealing at 55 °C for 30 s, and extension at 60 °C for 45 s. The gene *rpoD* was used as a control. See Table S2 for primer sequences.

#### *S*. *aureus*

500 ng of RNA was treated with TURBO DNase (Ambion/Life Technologies) according to the manufacturer’s instructions. The RNA was then converted into cDNA using the High Capacity cDNA Conversion Kit (Applied Biosystems/Life Technologies). Thermal cycling was performed using Maxima SYBR Green/ROX qRT-PCR Master Mix (Fermentas) in a Stratagene MX3000p with the following cycling conditions: Preincubation of 95 °C for 10 min followed by 45 cycles of 95 °C for 30 s; 60 °C for 60 s; 72 °C for 60 s. Upon completion, melting curve data was obtained. Data analysis was performed in the MxPro software version 4.1 (Stratagene). RNAIII and hla expression data was normalized to the expression of ileS, which was found to be stably expressed in all strains and conditions. See Table S2 for primer sequences.

### Genome analysis

RNA was purified according the descriptions above. Total RNA was checked for purity and intactness and sent for RNA sequencing at Beijing Genomic Institute. The resulting sequence data was analyzed in CLCbio’s Genomics Workbench v. 6.0.1 (QIAGEN). The reads were mapped back on the ≈3000 ORFs on the *S*. *aureus* NCTC 8325 reference genome (NC_007795.1) using the RPKM (Reads Per Kilobase transcript per Million reads) method as previously described^55^. Briefly, the number of reads mapping to every ORF are counted. For every ORF, this count is normalized according to the length of the ORF (per kb) and then normalized to the total number of reads in the sample (per million reads). This yields a quantitative absolute measure for the number of mRNAs per gene (the expression level) which is comparable between samples. The RNAseq data was submitted to the European nucleotide Archive ENA; http://www.ebi.ac.uk/ena.

To determine the reproducibility between the biological duplicates, scatter plots for each duplicate data sets from each of the three time points were made and the Pearson coefficient was over 0,97 in all cases, indicating good agreement between all duplicates (an example scatter plot is shown in Fig. S9 and all Pearson coefficients are listed in Table S3). To determine how many genes showed altered transcript levels in the presence of ajoene, all fold change values for each time point were sorted by value and plotted in separate charts (Fig. S10a–c). Furthermore, the differences between fold change values for all genes were visualized as shown in Fig. S10d‒f. Combined, Fig. S10 shows that the variation due to technical error and biological variation combined excluded drawing conclusions from fold changes smaller than twofold, and that four-fold changes should be approached with caution.

### Northern blot

Five µg of RNA extracted from 8325-4 cultures after 60′ (late exponential) and 240′ minutes (early stationary) of growth post ajoene treatment (see previous description) was loaded onto a 1% agarose gel and separated in 10 mM sodium phosphate buffer as described previously^56^. RNA was transferred to a positively charged nylon membrane (Boehringer Mannheim) by capillary blotting. Hybridization was performed according to^56^ using gene-specific probes that had been labeled with [32 P]dCTP using the Ready-to-Go DNA-labeling beads (Amersham Biosciences). Internal fragments of the *hla* or the *rnalll* gene were used as template in the labeling reactions. See Table S2 for primer sequences.

### Statistics

Statistical analyses were performed with GraphPad Prism version 6.0, GraphPad software (San Diego California, USA). To compare differences in biofilm and eDNA between ajoene treated and untreated a unpaired *t*-test was used. *p*-values < 0.05 were considered significant.

### Data availability

All data generated or analysed during this study are included in this published article (and its Supplementary Information file).

## Electronic supplementary material

Supplementary file
