# Supplementary material for: A broad range quorum sensing inhibitor working through sRNA inhibition
Source: Sci Rep. 2017 Aug 29;7:9857. doi: 10.1038/s41598-017-09886-8 (PMC5575346; doi:10.1038/s41598-017-09886-8)
Supplement: Supplementary file 1 — Supplementary file [file 41598_2017_9886_MOESM1_ESM.pdf]

Supplementary file

A broad range quorum sensing inhibitor working through sRNA inhibition

Tim H Jakobsen[1#], Anders N Warming[2], Rebecca M Vejborg[1], Joana A Moscoso[3], Marc Stegger[4], Frederik Lorenzen[1], Morten Rybtke[1], Jens Bo Andersen[1], Rico Petersen[5], Paal Skytt Andersen[4], Thomas E Nielsen[1,5,6], Tim Tolker-Nielsen[1], Alain Filloux[3], Hanne Ingmer[2], Michael Givskov[1,6]

[1]Costerton Biofilm Center, Department of Immunology and Microbiology, University of Copenhagen, Denmark.
[2]Department of Veterinary Pathobiology, University of Copenhagen, Copenhagen, Denmark.
[3]Centre for Molecular Bacteriology and Infection, Division of Cell and Molecular Biology, Imperial College London, London, UK.
[4]Department of Microbiology and Infection Control, Statens Serum Institut, Copenhagen, Denmark
[5]Department of Chemistry, Technical University of Denmark, Kgs. Lyngby, Denmark
[6]Singapore Centre on Environmental Life Sciences Engineering, Nanyang Technological University, Singapore.

[#]Corresponding author
Department of Immunology and Microbiology, University of Copenhagen, DK-2200 Copenhagen, Denmark. Phone: +4522542727. E-mail: tholm@sund.ku.dk.

Supplementary figures

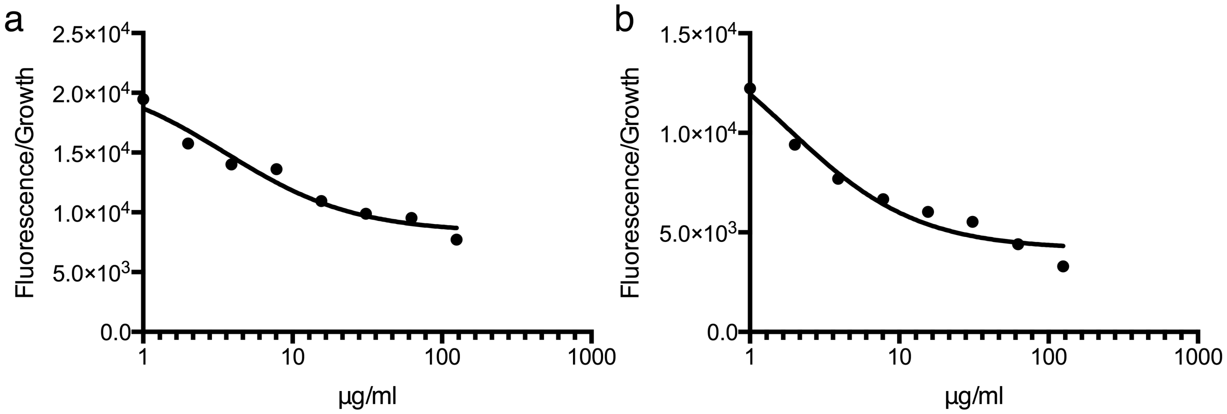

Figure S1. The half-maximal inhibitory concentration (IC_{50}) for ajoene tested with the following monitor strains: *rsmY-gfp* (**a**) and *rsmZ-gfp* (**b**). IC_{50} values were calculated with the software PRISM (GraphPad®).

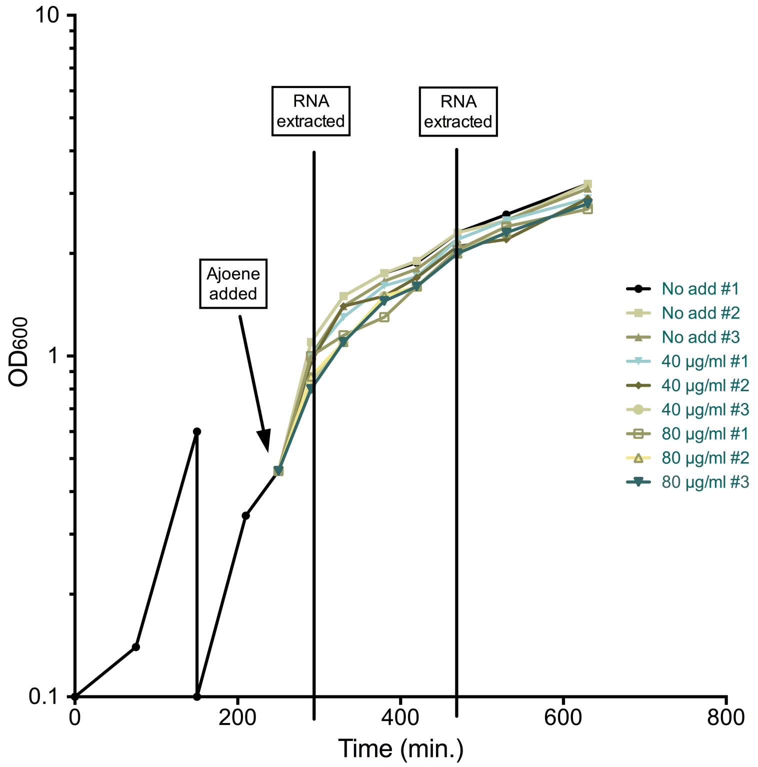

Figure S2. Growth of *P. aeruginosa*, PAO1 with and without ajoene. Ajoene tested in following concentrations; 40 µg/ml and 80 µg/ml. Samples for qRT-PCR were collected at late exponential growth phase (300 minutes) and early stationary growth phase (450 minutes). No add: No addition of ajoene.

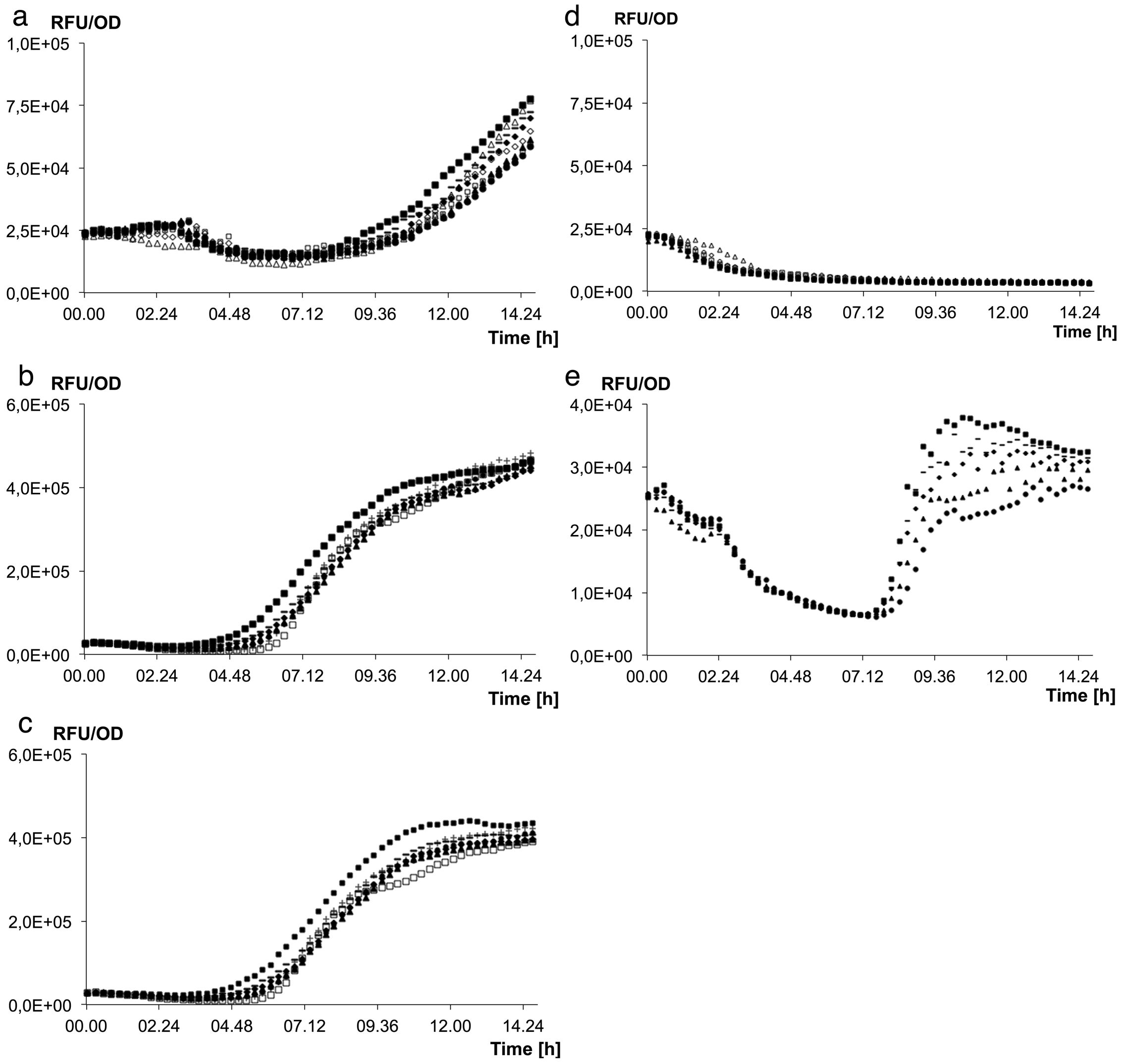

Figure S3. Regulation of *lasB* expression in Δ*rsmYZ,* Δ*rsmY,* Δ*rsmZ,* Δ*gacA* and Δ*rsmA* mutants by ajoene. The GFP expression/cell density (RFU/OD) of a *lasB-gfp* translational fusion in following background strains; *rsmYZ* (**a**), *rsmY* (**b**), and *rsmZ* (**c**), *gacA* (**d**) and rsmA (**e**) tested with following concentrations of ajoene: △125 µg/ml ◇62,5 µg/ml □31,25 µg/ml +15,6 µg/ml ●7,8 µg/ml▲3,9 µg/ml ◆2 µg/ml −1 µg/ml ■ No addition of ajoene. The experiments were done in triplicate.

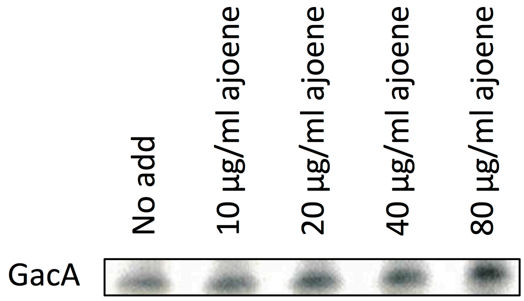

Figure S4. Changes in phosphorylation of GacA after treatment with following concentrations of ajoene; 10, 20, 40 and 80 µg/ml measured with Phos-tag gel combined with Western blot. No add: No addition of ajoene.

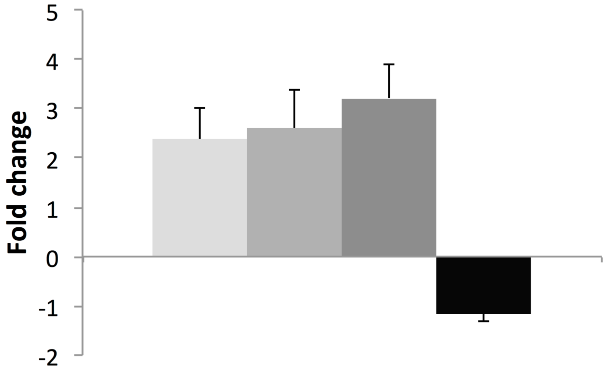

Figure S5. Regulation of following non-coding sRNAs, *phrS* (light grey), *prrF* (medium grey) and *prrH* (dark grey) as well as the housekeeping gene, *rpoD* (black) by ajoene in *P. aeruginosa*. qRT-PCR measurements of fold changes at early stationary growth phase (OD_{600} of 2.2). The data represent average of three individual experiments. Error bars are means ± SDs.

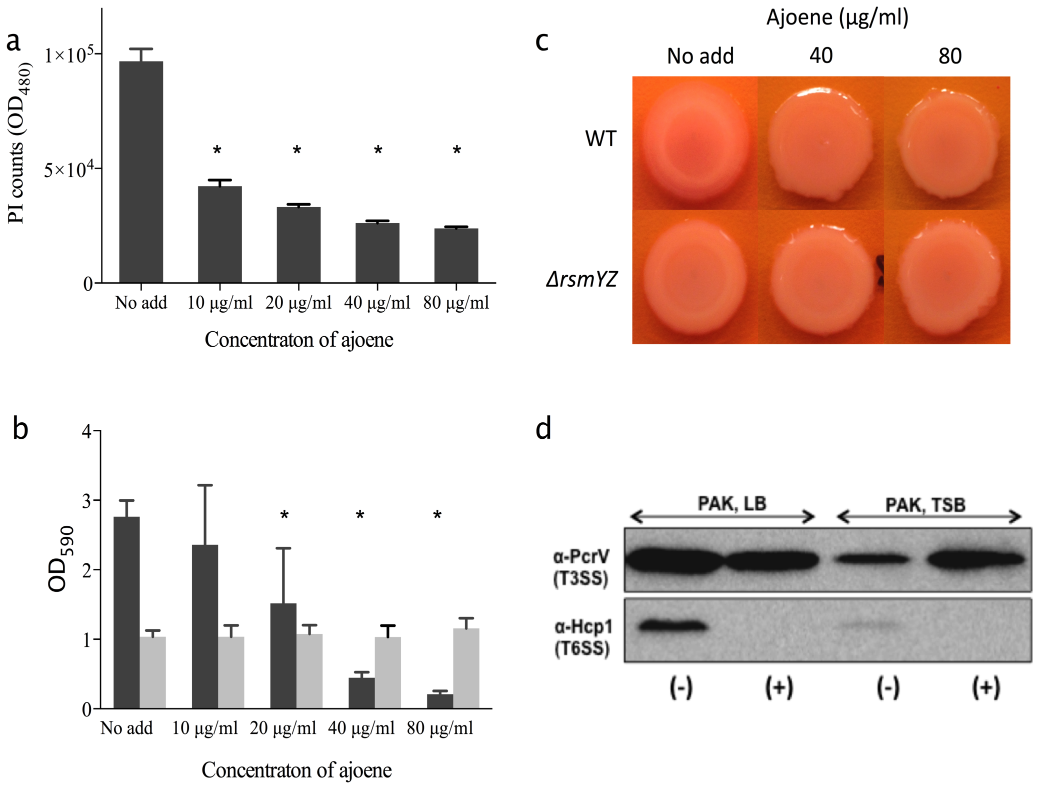

Figure S6. Phenotypic traits regulated by ajoene. **a**) Production of eDNA with and without different non-growth inhibiting concentrations of ajoene. The experiments were done in triplicate. There was a significant difference between untreated cultures and all cultures treated ajoene (*, P < 0.0001). No add: No addition of ajoene. PI: Propidium Iodide. Error bars are means ± SDs. **b**) Production of static biofilm by a *wt* (dark grey) and a *gacA* mutant (light grey) with and without ajoene supplemented growth medium. The experiments were done in triplicate. There was a significant difference between untreated cultures and cultures treated with 20 µg/ml to 80 µg/ml ajoene (*, P < 0.0001). No add: No addition of ajoene. Error bars are means ± SDs. **c**) Production of exopolysaccharide by a WT and *rsmYZ* mutant visualized by bacterial colony staining on Congo red-containing agar plates with and without ajoene added. No add: No addition of ajoene. **d**) Western blot of T3SS and T6SS without and with 80 µg/ml ajoene. Production of various secretion system components were detected by using

antibodies directed against PcrV (T3SS) and Hcp1 (T6SS) in both LB and TSB medium. (-) no ajoene added, (+) ajoene added.

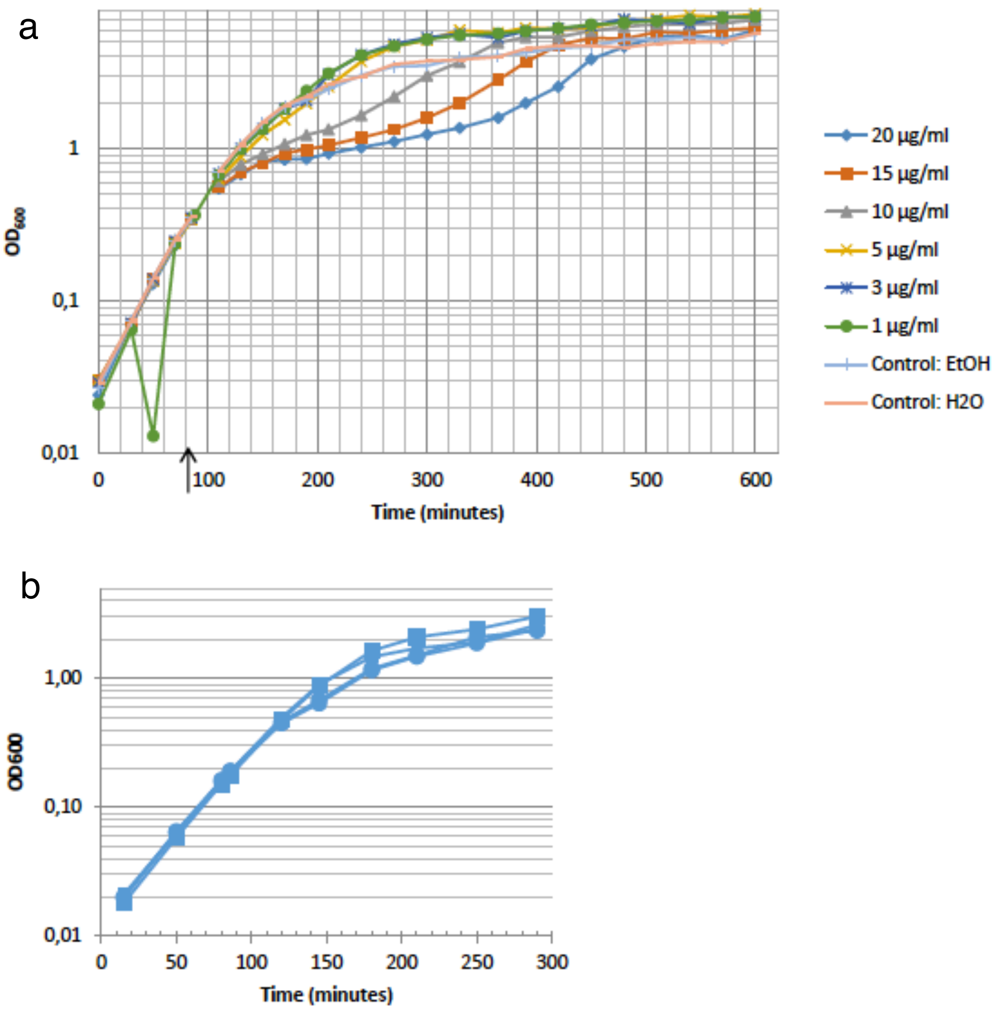

Figure S7. Growth of *S. aureus* 8325-4 with and without ajoene. **a)** Growth of *S. aureus* 8325-4 in the presence of increasing concentrations of ajoene. Ajoene is added at 90 minutes at an OD_{600} of 0.4 as indicated by the arrow. **b)** Growth of *S. aureus* 8325-4 in the presence of 5 µg/ml ajoene. Squares represent cultures treated with DMSO, circles represent cultures treated with 5 µg/ml ajoene.

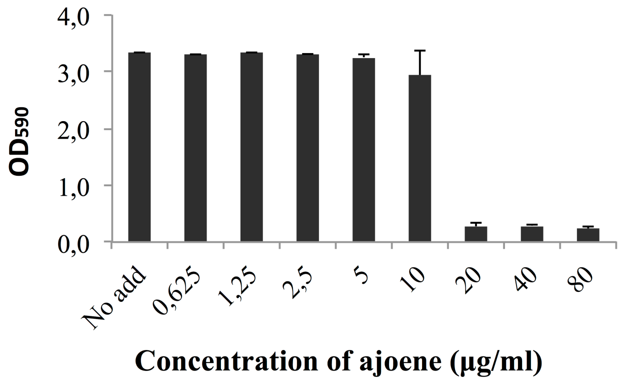

Figure S8. Production of static biofilm by *S. aureus* 8325-4 without and with ajoene supplemented growth medium in two-fold dilution from 80 to 0.625 µg/ml. There was no difference in biofilm formation with non-growth inhibitory concentrations of ajoene. The experiments were done in triplicate. No add: No addition of ajoene. Error bars are means ± SDs.

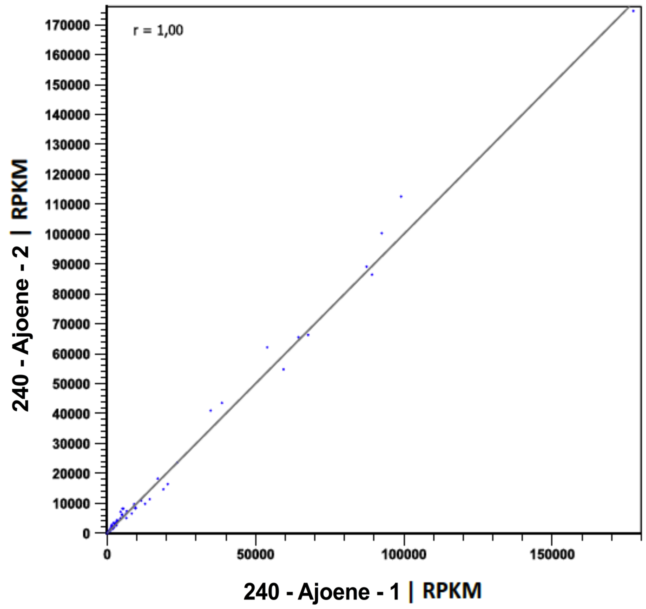

Figure S9. Scatter plot showing a comparison of RPKM (Reads Per Kilobase transcript per Million reads) between the replicate samples of ajoene at 240 minutes.

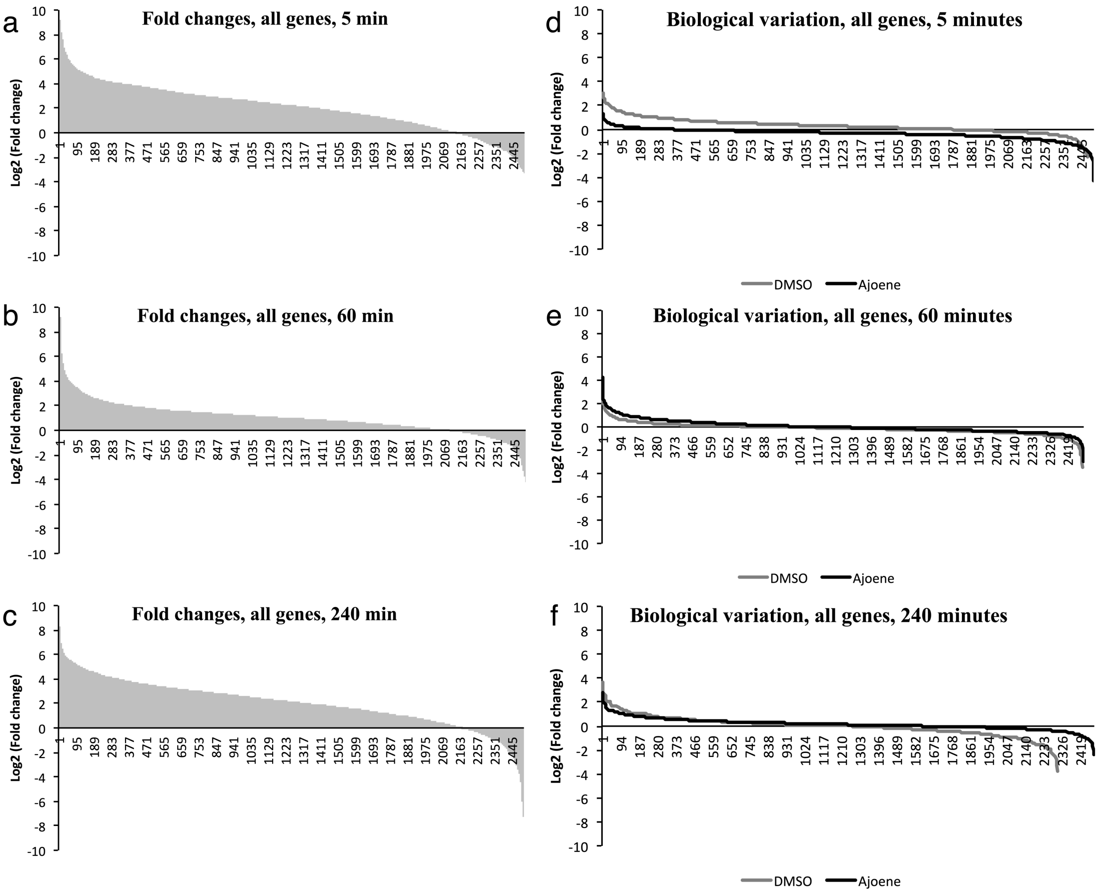

Figure S10. Global characteristics of the RNA sequencing data set. **a-c**) Global fold changes at 5’, 60’ and 240’ minutes sorted by magnitude. **d-f**) The variation between biological replicates at 5’, 60’ and 240’ minutes. On all charts, the Y-axis shows the log base 2 of the fold change.

Supplementary tables

Table S1 Transcript level and calculated fold change. Addition of 5 µg/ml ajoene at each of the three time points, 5', 60' and 240' minutes.

Gene	Locus tag (SAOUHSC_)	RPKM 5 min untreated	RPKM 5 min Ajoene	RPKM 60 min untreated	RPKM 60 min Ajoene	RPKM 240 min untreated	RPKM 240 min Ajoene	Fold change 5 min	Fold change 60 min	Fold change 240 min
**Selected virulence indicators**										
RNAIII	02260	14,30	21,75	69,00	5,42	4517,58	67,16	1,521	0,079	0,015
hla	01121	3,00	13,61	5,10	3,23	1259,99	12,75	4,531	0,634	0,010
spa	00069	113,97	369,07	210,60	217,95	95,94	462,89	3,238	1,035	4,825
**The agr operon**										
agrA	02265	27,36	65,77	36,73	34,87	147,14	88,52	2,404	0,949	0,602
agrB	02261	11,50	84,75	46,46	28,09	84,26	41,19	7,367	0,605	0,489
agrC	02264	8,51	43,22	20,58	16,70	57,81	26,71	5,081	0,811	0,462
agrD	02262	4,13	14,81	13,61	10,29	18,37	10,81	3,584	0,756	0,588
**Other pleiotropic response regulators**										
rot	01879	5,99	16,27	7,64	5,16	9,83	7,86	2,718	0,676	0,800
codY	01228	101,12	138,27	72,65	152,69	86,86	62,24	1,367	2,102	0,717
sarA	00620	49,61	590,16	28,40	130,61	1,02	10,52	11,896	4,599	10,310
saeS	00714	19,32	59,65	25,35	48,55	15,43	36,61	3,088	1,915	2,372
saeR	00715	13,48	85,61	33,67	47,35	13,06	32,49	6,351	1,406	2,488
sarS	00070	34,28	36,89	33,53	40,53	2,05	311,12	1,076	1,209	152,112
sarR	02566	3,88	11,16	4,96	2,67	0,55	4,95	2,877	0,539	9,077
ccpA	01850	141,45	211,85	147,03	113,30	41,34	135,34	1,498	0,771	3,274
σB	02298	78,12	179,54	61,23	146,68	11,51	59,09	2,298	2,396	5,134
**Proteases and lipases**										
V8 protease	00988	4,47	36,85	6,83	13,15	2273,96	9,73	8,248	1,925	0,004
splB	01941	0,53	10,40	1,33	0,99	226,91	0,98	19,524	0,748	0,004
splF	01935	1,47	7,26	2,53	2,00	346,23	2,24	4,944	0,789	0,006
splA	01942	1,08	9,16	0,41	1,17	190,61	1,45	8,477	2,876	0,008
splE	01936	1,07	14,99	2,01	3,60	378,23	3,68	14,051	1,795	0,010
sspB	00987	6,94	10,92	5,85	9,42	1341,02	13,68	1,575	1,611	0,010
splD	01938	0,53	9,28	0,93	2,88	233,58	2,69	17,362	3,089	0,011
splC	01939	0,81	12,80	0,80	2,31	199,37	2,55	15,755	2,893	0,013
sspC	00986	4,96	10,59	7,56	8,18	945,81	14,49	2,135	1,082	0,015
Aureolysin	02971	13,27	62,54	21,15	19,26	54,79	7,29	4,713	0,910	0,133
Lipase	03006	1,14	20,10	2,21	7,78	216,83	4,85	17,676	3,525	0,022
Lipase	00300	18,88	54,14	24,48	16,46	120,62	25,07	2,867	0,672	0,208

Table S1 continued

Gene	Locus tag (SAOUHSC_)	RPKM 5 min untreated	RPKM 5 min Ajoene	RPKM 60 min untreated	RPKM 60 min Ajoene	RPKM 240 min untreated	RPKM 240 min Ajoene	Fold change 5 min	Fold change 60 min	Fold change 240 min
**Autolysins**										
sle1	00427	48,55	328,72	87,91	521,06	3,44	79,56	6,771	5,927	23,149
atl	00994	23,25	267,60	48,50	185,34	3,45	44,57	11,510	3,822	12,907
**Autolysis regulators**										
cidC	02849	44,53	219,78	41,09	104,07	0,56	53,76	4,936	2,533	95,782
cidB	02850	36,67	367,61	61,47	218,67	0,55	68,42	10,026	3,558	124,323
cidA	02851	22,14	226,68	58,17	139,31	0,00	43,70	10,241	2,395	-
cidR	02852	50,16	101,91	39,85	62,98	1,86	48,77	2,031	1,580	26,219
lrgA	00232	0,86	6,44	2,16	0,92	2,15	2,76	7,474	0,427	1,285
lrgB	00233	0,56	8,27	1,09	2,64	32,99	0,86	14,886	2,413	0,026
**Phenol soluble modulins**										
PSM-alpha	00411	2,96	79,97	3,72	20,74	0,76	0,00	26,976	5,574	0,000
PSM-beta-1	01035	38,19	247,13	50,63	126,03	4,82	26,87	6,471	2,489	5,576
PSM-beta-2	01036	29,60	408,70	69,65	249,14	2,21	37,46	13,807	3,577	16,958
**Other cell-surface-associated proteins**										
clfA	00812	7917,65	5043,54	5961,16	14839,22	18235,83	17649,61	0,637	2,489	0,968
clfB	02963	35,69	913,28	40,58	484,48	2,80	129,83	25,587	11,939	46,341
coa	00192	7,95	21,80	7,28	12,04	3,84	12,44	2,742	1,653	3,238
fnbA	01175	32,08	190,36	32,66	94,02	5,00	24,48	5,934	2,878	4,898
fnbB	02802	4,20	359,73	6,62	82,82	0,81	13,14	85,705	12,512	16,263
icaA	03002	10,84	116,50	38,96	21,91	0,31	1,24	10,745	0,562	3,977
icaB	03004	21,24	62,42	25,50	22,99	4,44	22,70	2,939	0,902	5,117
icaC	03005	16,75	23,30	16,40	22,17	1,28	27,88	1,391	1,352	21,789
**Regulators of cell envelope turnover and charge**										
tagO	00762	5,39	49,43	8,81	21,48	0,81	5,09	9,173	2,437	6,241
vraF	00667	13,38	91,06	24,68	42,83	6,46	24,91	6,805	1,735	3,855
vraG	00668	21,66	46,04	18,18	35,77	3,16	23,85	2,125	1,968	7,542
yycF/walR	00020	25,90	346,78	62,74	155,52	13,71	35,40	13,390	2,479	2,582
yycG/walK	00021	13,93	180,88	33,50	84,29	3,97	14,74	12,985	2,516	3,715
graR	00665	4,71	59,01	10,38	33,39	4,43	9,28	12,527	3,217	2,097
graS	00666	21,21	79,16	24,71	63,83	10,00	32,66	3,732	2,583	3,266
graX	00664	10,75	222,75	25,86	104,96	6,05	21,09	20,723	4,059	3,487
mprF	01359	11,92	30,04	13,28	19,98	8,75	24,83	2,519	1,505	2,838

Table S1 continued

Gene	Locus tag (SAOUHSC_)	RPKM 5 min untreated	RPKM 5 min Ajoene	RPKM 60 min untreated	RPKM 60 min Ajoene	RPKM 240 min untreated	RPKM 240 min Ajoene	Fold change 5 min	Fold change 60 min	Fold change 240 min
**The dlt operon**										
dltX	00868	1929,50	1851,31	1801,10	3430,62	20,73	5411,17	0,959	1,905	261,048
dltA	00869	2030,94	2339,79	2100,05	3403,94	15,02	4763,44	1,152	1,621	317,150
dltB	00870	392,31	358,80	291,63	607,16	3,64	615,25	0,915	2,082	169,182
dltC	00871	422,38	411,20	295,62	714,86	6,91	599,78	0,974	2,418	86,776
dltD	00872	460,33	626,15	336,18	915,43	14,10	728,14	1,360	2,723	51,636
**SaPI3**										
-	01935	1,47	7,26	2,53	2,00	346,23	2,24	4,944	0,789	0,006
-	01936	1,07	14,99	2,01	3,60	378,23	3,68	14,051	1,795	0,010
-	01938	0,53	9,28	0,93	2,88	233,58	2,69	17,362	3,089	0,011
-	01939	0,81	12,80	0,80	2,31	199,37	2,55	15,755	2,893	0,013
-	01941	0,53	10,40	1,33	0,99	226,91	0,98	19,524	0,748	0,004
-	01942	1,08	9,16	0,41	1,17	190,61	1,45	8,477	2,876	0,008
-	01944	2,74	47,13	3,22	6,40	2177,84	4,09	17,183	1,990	0,002
**Selected secreted toxins**										
lukD	01954	0,39	4,44	0,39	0,73	1,18	1,15	11,268	1,880	0,972
lukE	01955	1,59	6,52	1,38	1,02	1,72	4,59	4,093	0,742	2,673
ear	01944	2,74	47,13	3,22	6,40	2177,84	4,09	17,183	1,990	0,002
eta (exfoliative toxin A)	01133	26,33	180,54	26,42	69,81	13,77	43,03	6,857	2,643	3,124
**All proteins containing enterotoxin domains**										
SAOUHSC_00354	00354	0,64	4,20	0,78	2,20	0,16	1,33	6,597	2,810	8,569
SAOUHSC_00383	00383	0,85	5,99	1,27	3,50	0,28	1,03	7,032	2,761	3,702
SAOUHSC_00384	00384	0,69	4,80	0,97	2,08	0,14	0,43	7,004	2,154	3,060
SAOUHSC_00386	00386	5,38	12,73	4,75	9,41	3,25	8,19	2,366	1,980	2,523
SAOUHSC_00389	00389	1,87	5,22	1,86	3,68	0,31	2,75	2,799	1,975	8,958
SAOUHSC_00390	00390	2,73	1,35	1,91	2,34	0,13	1,00	0,493	1,227	7,442
SAOUHSC_00391	00391	34,12	18,11	35,71	29,55	19,26	41,48	0,531	0,828	2,153
SAOUHSC_00392	00392	2,62	2,87	2,21	2,67	2,90	3,20	1,096	1,208	1,104
SAOUHSC_00393	00393	2,34	1,36	2,20	3,10	1,52	1,75	0,581	1,412	1,149
SAOUHSC_00394	00394	2,90	5,05	2,06	6,23	0,55	2,47	1,742	3,028	4,477
SAOUHSC_00395	00395	25,28	20,95	19,93	26,93	44,16	22,97	0,829	1,351	0,520
SAOUHSC_00399	00399	3,99	3,78	3,26	1,53	1,71	2,40	0,947	0,469	1,402
SAOUHSC_01127	01127	0,13	11,52	1,19	1,99	0,13	1,39	85,766	1,677	10,272
Enterotoxin type A	01705	2,73	22,23	6,26	15,96	0,42	3,17	8,141	2,550	7,613

Table S1 continued

Gene	Locus tag (SAOUHSC_)	RPKM 5 min untreated	RPKM 5 min Ajoene	RPKM 60 min untreated	RPKM 60 min Ajoene	RPKM 240 min untreated	RPKM 240 min Ajoene	Fold change 5 min	Fold change 60 min	Fold change 240 min
**Hemolysins**										
alpha-hemolysin	01121	3,00	13,61	5,10	3,23	1259,99	12,75	4,531	0,634	0,010
beta-hemolysin	02240	20,25	31,76	27,92	23,20	1634,76	186,08	1,569	0,831	0,114
RNAIII / delta-hemolysin	02260	14,30	21,75	69,00	5,42	4517,58	67,16	1,521	0,079	0,015
gamma-hemolysin	02708	1,14	5,22	1,34	1,89	12,99	1,87	4,573	1,412	0,144
**ESAT-6 secretion pathway**										
esxA	00257	563,71	196,08	906,55	21,72	1100,77	811,64	0,348	0,024	0,737
esaA	00258	1084,92	133,70	1399,54	60,21	1461,88	1485,86	0,123	0,043	1,016
essA	00259	3955,79	458,41	4740,56	251,79	6579,47	5517,42	0,116	0,053	0,839
esaB	00260	2473,28	206,20	2782,98	118,49	3536,46	3030,31	0,083	0,043	0,857
essB	00261	4750,25	468,34	5337,11	342,39	9010,11	5662,48	0,099	0,064	0,628
essC	00262	3324,96	415,61	3984,19	223,36	4279,58	3767,99	0,125	0,056	0,880
esaC	00264	1345,00	258,95	1615,64	102,36	1003,80	1281,91	0,193	0,063	1,277
esxB	00265	847,94	159,40	1018,56	44,74	610,87	679,18	0,188	0,044	1,112
esaE	00266	889,51	154,50	1032,39	68,06	623,49	840,03	0,174	0,066	1,347
esaF	00267	583,73	129,68	713,33	46,51	475,10	464,19	0,222	0,065	0,977
esaD	00268	700,83	210,58	827,04	73,28	622,67	553,39	0,300	0,089	0,889
**The *ure* operon**										
Putative urea transporter	02557	1,19	24,38	1,62	12,86	0,43	7,21	20,497	7,963	16,648
ureA	02558	1,28	264,62	0,63	120,11	3,18	17,39	206,895	189,956	5,464
ureB	02559	2,57	474,34	3,27	197,79	9,16	42,63	184,374	60,473	4,656
ureC	02561	1,35	278,71	2,24	98,36	0,61	18,50	206,601	43,965	30,145
ureE	02562	1,50	162,61	1,91	62,43	0,85	16,96	108,717	32,732	19,913
ureF	02563	1,40	93,98	2,22	34,82	0,99	10,58	67,196	15,658	10,694
ureG	02564	2,85	128,95	8,74	53,20	1,42	24,94	45,308	6,089	17,519
ureD	02565	4,02	62,44	6,07	36,82	1,04	18,53	15,532	6,061	17,776
**TCA cycle enzymes**										
sucB	01416	250,73	113,83	352,01	243,55	8818,40	357,92	0,454	0,692	0,041
sucA	01418	93,96	72,04	147,73	73,05	3591,61	117,74	0,767	0,495	0,033
SAOUHSC_01614	01614	19,15	147,74	34,96	103,57	5,28	22,51	7,715	2,963	4,264
SAOUHSC_01043	01043	96,37	111,20	73,18	115,27	41,25	108,37	1,154	1,575	2,627
SAOUHSC_01266	01266	27,59	120,09	25,77	49,89	1,37	15,73	4,352	1,936	11,454
SAOUHSC_01267	01267	36,30	88,35	29,43	48,18	1,67	16,43	2,434	1,637	9,836
SAOUHSC_01802	01802	11,48	134,13	23,70	61,53	30,16	8,78	11,686	2,596	0,291
SAOUHSC_01347	01347	11,44	56,94	15,49	31,58	13,77	7,78	4,977	2,038	0,565
SAOUHSC_01801	01801	11,60	46,61	13,61	26,44	21,42	9,20	4,019	1,942	0,430
sucC	01216	24,77	135,40	45,39	75,17	29,09	19,44	5,467	1,656	0,668
SAOUHSC_01218	01218	19,48	65,02	25,96	37,97	27,24	13,50	3,339	1,462	0,496
SAOUHSC_01103	01103	6,87	43,77	16,39	10,26	7,85	3,99	6,368	0,626	0,509
sdhA	01104	12,45	76,77	26,44	19,64	25,07	9,82	6,166	0,743	0,392

sdhB	01105	10,27	28,78	14,00	11,24	24,06	8,90	2,803	0,803	0,370

Table S2: Strains, plasmids and primers used in this study.

Strain, plasmid and primer	Characteristics and sequence (5'→3')	Reference
***S. aureus* strains**		
8325-4	Wild-type *S. aureus*	[1]
RC203	*spa::lacZ*, Erm^R	[2]
SH101F7	*RNAIII::lacZ*, Erm^R	[3]
***P. aeruginosa* strains**		
PAO1	Wild-type *P. aeruginosa*	www.pseudomonas.med.ecu.edu PAO0001
Δ*rsmYZ*	*rsmYZ* mutant	[4]
Δ*rsmY*	*rsmY* mutant	[4]
Δ*rsmZ*	*rsmZ* mutant	[5]
Δ*gacA*	*gacA*::Ω-Sm/Sp; Sm/Sp^r	[6]
Δ*rsmA*	*rsmA* mutant	[7]
Δ*retS*	*retS* mutant	This study
lasB-gfp	*lasB-gfp*(ASV) translational fusion, Ap^R Gm^R	[8]
PAO098	*rsmZ-gfp*mut3* transcriptional fusion, Gm^R, Ap^R	This study
PAO099	*rsmY-gfp*mut3* transcriptional fusion, Gm^R, Ap^R	This study
PAO101	Δ*rsmYZ, lasB-gfp*(ASV), Ap^R Gm^R	This study
PAO102	Δ*rsmY, lasB-gfp*(ASV), Ap^R Gm^R	This study
PAO103	Δ*rsmZ, lasB-gfp*(ASV), Ap^R Gm^R	This study
PAO104	Δ*rsmA, lasB-gfp*(ASV), Ap^R Gm^R	This study
PAO105	Δ*gacA, lasB-gfp*(ASV), Ap^R Gm^R	This study
PAO106	Δ*retS, rsmZ-gfp*mut3*, Gm^R, Ap^R	This study
PAO107	Δ*retS, rsmY-gfp*mut3*, Gm^R, Ap^R	This study
**Plasmids**		
pMHLAS	pMH391 carrying P*lasB-gfp*(ASV), Ap^R Gm^R	[8]
pMHRA	pMH391 carrying P*rhlA-gfp*(ASV), Ap^R Gm^R	[9]
pMP220*rsmZ-lacZ*	pMP220 carrying a *rsmZ-lacZ* transcriptional fusion, Tc^R	[10]
pMP220*rsmY-lacZ*	pMP220 carrying a *rsmY-lacZ* transcriptional fusion, Tc^R	[10]
pMH305	Broad-host-range vector, pUCP22Not-RBSII-*gfp*(Mut3)-T$_0$-T$_1$,Gm^R, Ap^R	[11]
pJBA25	pUC18Not-RBSII-*gfp*mut3*-T$_0$-T$_1$, Ap^R	[12]
pDONR221	Gateway donor vector, Kan^R Cam^R	Invitrogen
pEX18ApGW	Gateway compatible gene replacement vector, Amp^R Cam^R	[13]
pPS856	Source of the FRT-flanked gentamicin resistance cassette, Amp^R	[14]

	Gm^R	
pFLP2	Flp recombinase expression vector	[14]
pΔretS	retS deletion vector, Amp^R Gm^R	This study
pRV59_1	pMH305 carrying *rsmZ-gfp*mut3* transcriptional fusion, Gm^R, Ap^R	This study
pRV60_1	pMH305 carrying *rsmY-gfp*mut3* transcriptional fusion, Gm^R, Ap^R	This study
**Primers**		
rsmY forward[a]	GCGCCAAAGACAATACGGAA	This study
rsmY reverse[a]	CTCTATCCTGACATCCGTGCT	This study
rsmZ forward[a]	CCCACTCTTCAGTCCCTCGT	This study
rsmZ reverse[a]	AACACGCAACCCCGAAGGAT	This study
rpoD forward	ACAAGATCCGCAAGGTACTGAAG	[15]
rpoD reverse	CGCCCAGGTGCGAATC	[15]
phrS forward[a]	CGTTCTCGCAGGGATTCTTA	This study
phrS reverse[a]	CCTTGCGTGCTCTGTGTATC	This study
prrF forward[a]	AACTGGTCGCGAGATCAGC	This study
prrF reverse[a]	CCGTGATTAGCCTGATGAGGAG	This study
prrH forward[a]	CGATGGAATGAATGAGAACCG	This study
prrH reverse[a]	TTGGTCTCTCAGCTTACCTG	This study
retS_UpF-GWL[a]	TACAAAAAAGCAGGCTGTGCTGGACGAAGTGATCTACCC	This study
retS _UpR-GM[a]	TCAGAGCGCTTTTGAAGCTAATTCGGAGTAGTCCTATGGC GATCCGAA	This study
retS _DnF-GM[a]	AGGAACTTCAAGATCCCCAATTCGTGAGCTGATCGCCTAC TGGGTC	This study
retS _DnR-GWR[a]	TACAAGAAAGCTGGGTAAGAGGATGAAGATGTAGCGGCC	This study
Gm-F	CGAATTAGCTTCAAAAGCGCTCTGA	[13]
Gm-R	CGAATTGGGGATCTTGAAGTTCCT	[13]
GW-attB1	GGGGACAAGTTTGTACAAAAAAGCAGGCT	[13]
GW-attB2	GGGGACCACTTTGTACAAGAAAGCTGGGT	[13]
spa forward[b]	CAAACGGCACTACTGCTGAC	This study
spa reverse[b]	CATGGTTTGCTGGTTGCTTC	This study
hla forward[b]	AGCGAAGTCTGGTGAAAACC	This study
hla reverse[b]	CGGTATATGGCAATCAACTTT	This study
rnaIII forward[b]	GCACTGAGTCCAAGGAAACTAAC	This study
rnaIII reverse[b]	AAGCCATCCCAACTTAATAACC	This study
iles forward[b]	ACATACAGCACCAGGTCACG	This study
iles reverse[b]	CGCCTTCTTCAGTAAATACACC	This study

[a]*P. aeruginosa* PAO1, GeneBank accession no. NC_2516.2 used to design primers.
[b]*S. aureus* 8325-4, GeneBank accession no. CP000253 used to design primers.

Table S3. Pearson coefficients from all Scatter plots.

Scatter plot	Pearson coefficient
5-DMSO-1 vs 5-DMSO-2	1.00
5-ajoene-1 vs 5-ajoene-2	0.97
60-DMSO-1 vs 60-DMSO-2	0.99
60-ajoene-1 vs 60-ajoene-2	0.96
240-DMSO-1 vs 240-DMSO-2	0.99
240-ajoene-1 vs 240-ajoene-2	1.00

Supplementary material and methods

Static biofilm formation

P. aeruginosa: O.N. cultures of *wt* PAO1 and Δ*gacA* were diluted to an OD_{450} of 0.02 in fresh AB minimal medium supplemented with 0.5% (wt/vol) glucose and 0.5% (wt/vol) Casamino acids and then incubated in 96 well polystyrene microtiter plates (Sterilin® Ltd) at 37°C for 16 hours with and without different concentrations of ajoene.

S. aureus: O.N. culture of *S. aureus* 8325-4 were diluted 1:100 in fresh TSB media and then incubated in 96 well polystyrene plates (Sterilin® Ltd) at 37°C for 24 hours with and without different concentrations of ajoene.

Media was removed and wells washed with 0,9% NaCl to remove non-adherent cells. Adherent cells were stained for 30 min. with 0.1% crystal violet (CV) (Sigma) solution and washed twice with 0.9% NaCl to remove non-bound CV. 96% EtOH were added to dissolve bound CV and formation of biofilm were measured using a microplate reader (Victor X, PerkinElmer) by determination of OD_{590}.

Extracellular DNA

O.N. culture of PAO1 was diluted to an OD_{600} of 0.001 in fresh AB minimal medium

supplemented with 0.5% (wt/vol) glucose and 0.05 mM propidium iodide and then incubated in 96 well polystyrene microtiter plates (Sterilin® Ltd) at 37°C for 16 hours with and without different concentrations of ajoene. Propidium iodide and growth was measured using a microplate reader (Victor X, PerkinElmer) by determination of OD_{480nm} and OD_{600nm}, respectively[16].

Exopolysaccharide production

Agar (1%) plates supplemented with tryptone (1%), Congo red (40 mg/ml) and ajoene in concentrations of 40 µg/ml and 80 µg/ml as well as one with no addition of ajoene were used. From O.N. cultures of *wt* PAO1 and a *rsmYZ* mutant 5 µl were added on top of the plates and incubated at RT for 5 days, where after, colony staining were visually inspected.

Western blot analysis of T3SS and T6SS

Detection of PcrV and Hcp1 in *P. aeruginosa* cell lysates was performed as described in Moscoso *et al.*[17]. Briefly, *P. aeruginosa* PAK strain was inoculated into LB or TSB medium to an OD_{600} of 0.1 and incubated at 37°C for 6 hours, shaking. Lysates were separated by SDS-PAGE using a 12% resolving gel. Primary antibodies, anti-Hcp1 and anti-PcrV were used at 1:500 and 1:1000 dilutions, respectively. The same membrane was probed with the two primary antibodies. No loading control was used but two independent experiments were performed.

Mobility shift detection of phosphorylated GacA

P. aeruginosa cultures were grown according to the description in the manuscript "*RNA preparation*" without and with ajoene (80 µg/ml). Samples were retrieved at early stationary phase (OD_{600} of 2.2). Lysates were separated by SuperSep Phos-tag 12.5 %

gel (Wako, Japan) and subsequently analyzed by Western blot with a primary anti-GacA antibody[18].

Supplementary material references

1 Novick, R. P. & Morse, S. I. In vivo transmission of drug resistance factors between strains of *Staphylococcus aureus*. *The Journal of experimental medicine* **125**, 45-59 (1967).

2 Chan, P. F. & Foster, S. J. The role of environmental factors in the regulation of virulence-determinant expression in *Staphylococcus aureus* 8325-4. *Microbiology* **144 (Pt 9)**, 2469-2479, doi:10.1099/00221287-144-9-2469 (1998).

3 Horsburgh, M. J. *et al.* sigmaB modulates virulence determinant expression and stress resistance: characterization of a functional rsbU strain derived from *Staphylococcus aureus* 8325-4. *Journal of bacteriology* **184**, 5457-5467 (2002).

4 Kay, E. *et al.* Two GacA-dependent small RNAs modulate the quorum-sensing response in *Pseudomonas aeruginosa*. *Journal of bacteriology* **188**, 6026-6033, doi:10.1128/JB.00409-06 (2006).

5 Heurlier, K. *et al.* Positive control of swarming, rhamnolipid synthesis, and lipase production by the posttranscriptional RsmA/RsmZ system in *Pseudomonas aeruginosa* PAO1. *Journal of bacteriology* **186**, 2936-2945 (2004).

6 Reimmann, C. *et al.* The global activator GacA of *Pseudomonas aeruginosa* PAO positively controls the production of the autoinducer N-butyryl-homoserine lactone and the formation of the virulence factors pyocyanin, cyanide, and lipase. *Molecular microbiology* **24**, 309-319 (1997).

7 Pessi, G. *et al.* The global posttranscriptional regulator RsmA modulates production of virulence determinants and N-acylhomoserine lactones in *Pseudomonas aeruginosa*. *Journal of bacteriology* **183**, 6676-6683, doi:10.1128/JB.183.22.6676-6683.2001 (2001).

8 Hentzer, M. *et al.* Inhibition of quorum sensing in *Pseudomonas aeruginosa* biofilm bacteria by a halogenated furanone compound. *Microbiology* **148**, 87-102 (2002).

9 Yang, L. *et al.* Computer-aided identification of recognized drugs as *Pseudomonas aeruginosa* quorum-sensing inhibitors. *Antimicrob Agents Chemother* **53**, 2432-2443, doi:AAC.01283-08 [pii] 10.1128/AAC.01283-08 (2009).

10 Bordi, C. *et al.* Regulatory RNAs and the HptB/RetS signalling pathways fine-tune *Pseudomonas aeruginosa* pathogenesis. *Mol Microbiol* **76**, 1427-1443, doi:10.1111/j.1365-2958.2010.07146.xMMI7146 [pii] (2010).

11 Rybtke, M. T. *et al.* Fluorescence-based reporter for gauging cyclic di-GMP levels in *Pseudomonas aeruginosa*. *Appl Environ Microbiol* **78**, 5060-5069, doi:10.1128/AEM.00414-12 AEM.00414-12 [pii] (2012).

12 Moller, S. *et al.* In situ gene expression in mixed-culture biofilms: evidence of metabolic interactions between community members. *Appl Environ Microbiol* **64**, 721-732 (1998).

13 Choi, K. H. & Schweizer, H. P. An improved method for rapid generation of unmarked *Pseudomonas aeruginosa* deletion mutants. *BMC microbiology* **5**, 30, doi:10.1186/1471-2180-5-30 (2005).
14 Hoang, T. T., Karkhoff-Schweizer, R. R., Kutchma, A. J. & Schweizer, H. P. A broad-host-range Flp-FRT recombination system for site-specific excision of chromosomally-located DNA sequences: application for isolation of unmarked *Pseudomonas aeruginosa* mutants. *Gene* **212**, 77-86 (1998).
15 Jakobsen, T. H. *et al.* Ajoene, a sulfur-rich molecule from garlic, inhibits genes controlled by quorum sensing. *Antimicrobial agents and chemotherapy* **56**, 2314-2325, doi:10.1128/AAC.05919-11 (2012).
16 Allesen-Holm, M. *et al.* A characterization of DNA release in *Pseudomonas aeruginosa* cultures and biofilms. *Mol Microbiol* **59**, 1114-1128, doi:10.1111/j.1365-2958.2005.05008.x (2006).
17 Moscoso, J. A., Mikkelsen, H., Heeb, S., Williams, P. & Filloux, A. The *Pseudomonas aeruginosa* sensor RetS switches type III and type VI secretion via c-di-GMP signalling. *Environmental microbiology* **13**, 3128-3138, doi:10.1111/j.1462-2920.2011.02595.x (2011).
18 Takeuchi, K. *et al.* Lon protease negatively affects GacA protein stability and expression of the Gac/Rsm signal transduction pathway in *Pseudomonas protegens*. *Environmental microbiology* **16**, 2538-2549, doi:10.1111/1462-2920.12394 (2014).
